# Supplementary material for: Investigation of Transmission and Evolution of PEDV Variants and Co-Infections in Northeast China from 2011 to 2022
Source: Animals (Basel). 2024 Jul 25;14(15):2168. doi: 10.3390/ani14152168 (PMC11311072; doi:10.3390/ani14152168)
Supplement: Supplementary file 1 [file animals-14-02168-s001.zip › Figure S1.docx]

**Figure S1.** Year-on-year comparison of the S protein modeling differences between 2011 and 2022 prevalent PEDV strains. (**a**) 2011 and 2012–PEDV strains; (**b**) 2012 and 2013–PEDV strains; (**c**) 2013 and 2014–PEDV strains; (**d**) 2014 and 2015–PEDV strains; (**e**) 2015 and 2016–PEDV strains; (**f**) 2016 and 2017–PEDV strains; (**g**) 2017 and 2018–PEDV strains; (**h**) 2018 and 2019–PEDV strains; (**i**) 2019 and 2020–PEDV strains; (**j**) 2020 and 2021–PEDV strains; (**k**) 2021 and 2022–PEDV strains;(**l**) CV777 and 2022–PEDV strains.
