# Supplementary material for: Investigation of Transmission and Evolution of PEDV Variants and Co-Infections in Northeast China from 2011 to 2022
Source: Animals (Basel). 2024 Jul 25;14(15):2168. doi: 10.3390/ani14152168 (PMC11311072; doi:10.3390/ani14152168)
Supplement: Supplementary file 1 [file animals-14-02168-s001.zip › Table S1.docx]

**Table S1.** The reference PEDV strains used in this study.

| Strain name※ | | Strain | | Countries | | Collection date | | GenBank accession no. | |  |  |  |
| --- | --- | --- | --- | --- | --- | --- | --- | --- | --- | --- | --- | --- |
| CV777 | | PEDV | | BEL | | 1988 | | AF353511 | |  |  |  |
| MEX/MICH/01/2013 | | PEDV | | MEX | | 2013 | | KY828999.1 | |  |  |  |
| MEX/JAL/03/2016 | | PEDV | | MEX | | 2016 | | KY828998.1 | |  |  |  |
| MEX/JAL/01/2016 | | PEDV | | MEX | | 2016 | | KY828997.1 | |  |  |  |
| MEX/MICH/02/2015 | | PEDV | | MEX | | 2015 | | KY828996.1 | |  |  |  |
| MEX/MICH/01/2015 | | PEDV | | MEX | | 2015 | | KY828995.1 | |  |  |  |
| MEX/GTO/02/2014 | | PEDV | | MEX | | 2014 | | KY828994.1 | |  |  |  |
| MEX/JAL/01/2014 | | PEDV | | MEX | | 2014 | | KY828993.1 | |  |  |  |
| MEX/SON/01/2014 | | PEDV | | MEX | | 2014 | | KY828992.1 | |  |  |  |
| MEX/VER/01/2014 | | PEDV | | MEX | | 2014 | | KY828991.1 | |  |  |  |
| 0100/3T | | PEDV | | Poland | | 2021 | | MZ216022.1 | |  |  |  |
| 25364/1 | | PEDV | | Poland | | 2021 | | MZ216021.1 | |  |  |  |
| 6706/1 | | PEDV | | Poland | | 2021 | | MZ216020.1 | |  |  |  |
| 0100/5P | | PEDV | | Poland | | 2021 | | MZ216018.1 | |  |  |  |
| 44176/2 | | PEDV | | Poland | | 2021 | | MZ216019.1 | |  |  |  |
| XJ1904-34 | | PEDV | | CHN | | 2021 | | OK642747.1 | |  |  |  |
| VN-TH15/HY/2015 | | PEDV | | USA | | 2015 | | KX982576.1 | |  |  |  |
| VN-K2/HY/2015 | | PEDV | | USA | | 2015 | | KX982573.1 | |  |  |  |
| VN385/TB/2014 | | PEDV | | USA | | 2014 | | KX982572.1 | |  |  |  |
| VN367/VP/2014 | | PEDV | | USA | | 2014 | | KX982571.1 | |  |  |  |
| VN344/HN/2014 | | PEDV | | USA | | 2014 | | KX982570.1 | |  |  |  |
| VN297/HB/2014 | | PEDV | | USA | | 2014 | | KX982569.1 | |  |  |  |
| VN292/HN/2014 | | PEDV | | USA | | 2014 | | KX982568.1 | |  |  |  |
| VN97/HN/2013 | | PEDV | | USA | | 2013 | | KX982561.1 | |  |  |  |
| VN232/HB/2013 | | PEDV | | USA | | 2013 | | KX982564.1 | |  |  |  |
| VN12/HY/2013 | | PEDV | | USA | | 2013 | | KX982557.1 | |  |  |  |
| VN02/HY/2013 | | PEDV | | USA | | 2013 | | KKX982554.1 | |  |  |  |
| VN01/HY/2013 | | PEDV | | USA | | 2013 | | KX982553.1 | |  |  |  |
| GD18 | | PEDV | | CHN | | 2015 | | KP698768.2 | |  |  |  |
| GD16 | | PEDV | | CHN | | 2015 | | KP698766.2 | |  |  |  |
| GD15 | | PEDV | | CHN | | 2015 | | KP698765.2 | |  |  |  |
| GD14 | | PEDV | | CHN | | 2015 | | KP698764.2 | |  |  |  |
| GD13 | | PEDV | | CHN | | 2015 | | KP698763.2 | |  |  |  |
| GD12 | | PEDV | | CHN | | 2015 | | KP698762.2 | |  |  |  |
| GD11 | | PEDV | | CHN | | 2015 | | KP698761.2 | |  |  |  |
| GD6 | | PEDV | | CHN | | 2015 | | KP698756.2 | |  |  |  |
| GD5 | | PEDV | | CHN | | 2015 | | KP698755.2 | |  |  |  |
| GD4 | | PEDV | | CHN | | 2015 | | KP698754.2 | |  |  |  |
| GD2 | | PEDV | | CHN | | 2015 | | KP698752.2 | |  |  |  |
| GD20 | | PEDV | | CHN | | 2015 | | KP698770.1 | |  |  |  |
| GD19 | | PEDV | | CHN | | 2015 | | KP698769.1 | |  |  |  |
| GD17 | | PEDV | | CHN | | 2015 | | KP698767.1 | |  |  |  |
| GD10 | | PEDV | | CHN | | 2015 | | KP698760.1 | |  |  |  |
| GD9 | | PEDV | | CHN | | 2015 | | KP698759.1 | |  |  |  |
| GD8 | | PEDV | | CHN | | 2015 | | KP698758.1 | |  |  |  |
| GD7 | | PEDV | | CHN | | 2015 | | KP698757.1 | |  |  |  |
| GD3 | | PEDV | | CHN | | 2015 | | KP698753.1 | |  |  |  |
| GD1 | | PEDV | | CHN | | 2015 | | KP698751.1 | |  |  |  |
| SX/YC/2019 | | PEDV | | CHN | | 2019 | | MT090140.1 | |  |  |  |
| HN/XC/2019 | | PEDV | | CHN | | 2019 | | MT090139.1 | |  |  |  |
| HN/KF/2019 | | PEDV | | CHN | | 2019 | | MT090138.1 | |  |  |  |
| HN/HX/2019 | | PEDV | | CHN | | 2019 | | MT090137.1 | |  |  |  |
| BP-2016 | | PEDV | | CHN | | 2016 | | LC496368.1 | |  |  |  |
| CH-ZJQZ-2-2016 | | PEDV | | CHN | | 2016 | | MG020555.1 | |  |  |  |
| CH-ZJQZ-1-2016 | | PEDV | | CHN | | 2016 | | MG020554.1 | |  |  |  |
| CH-ZJLS-1-2016 | | PEDV | | CHN | | 2016 | | MG020553.1 | |  |  |  |
| CH-ML-2-2016 | | PEDV | | CHN | | 2016 | | MG020552.1 | |  |  |  |
| CH-ML-1-2016 | | PEDV | | CHN | | 2016 | | MG020551.1 | |  |  |  |
| CH-JXXY-3-2016 | | PEDV | | CHN | | 2016 | | MG020550.1 | |  |  |  |
| CH-JXXY-2-2016 | | PEDV | | CHN | | 2016 | | MG020549.1 | |  |  |  |
| CH-JXXY-1-2016 | | PEDV | | CHN | | 2016 | | MG020548.1 | |  |  |  |
| CH-JXJJ-1-2016 | | PEDV | | CHN | | 2016 | | MG020547.1 | |  |  |  |
| CH-JSZJ-2-2016 | | PEDV | | CHN | | 2016 | | MG020546.1 | |  |  |  |
| CH-JSZJ-1-2016 | | PEDV | | CHN | | 2016 | | MG020545.1 | |  |  |  |
| CH-JSYC-1-2016 | | PEDV | | CHN | | 2016 | | MG020544.1 | |  |  |  |
| CH-HBMC-2-2016 | | PEDV | | CHN | | 2016 | | MG020543.1 | |  |  |  |
| CH-HBMC-1-2016 | | PEDV | | CHN | | 2016 | | MG020542.1 | |  |  |  |
| CH-AHXC-1-2016 | | PEDV | | CHN | | 2016 | | MG020541.1 | |  |  |  |
| CH-AHSZ-1-2016 | | PEDV | | CHN | | 2016 | | MG020540.1 | |  |  |  |
| CH-AHHX-1-2016 | | PEDV | | CHN | | 2016 | | MG020539.1 | |  |  |  |
| CH-AHBB-1-2016 | | PEDV | | CHN | | 2015 | | MG020538.1 | |  |  |  |
| YnP9 | | PEDV | | CHN | | 2017 | | MG334010.1 | |  |  |  |
| YnP8 | | PEDV | | CHN | | 2017 | | MG334009.1 | |  |  |  |
| YnP7 | | PEDV | | CHN | | 2017 | | MG334008.1 | |  |  |  |
| YnP6 | | PEDV | | CHN | | 2017 | | MG334007.1 | |  |  |  |
| YnP5 | | PEDV | | CHN | | 2017 | | MG334006.1 | |  |  |  |
| YnP4 | | PEDV | | CHN | | 2017 | | MG334005.1 | |  |  |  |
| YnP3 | | PEDV | | CHN | | 2017 | | MG334004.1 | |  |  |  |
| YnP2 | | PEDV | | CHN | | 2017 | | MG334003.1 | |  |  |  |
| YnP1 | | PEDV | | CHN | | 2017 | | MG334002.1 | |  |  |  |
| JMi-277/fSnorCo12 | | PEDV | | CHN | | 2015 | | KU363112.1 | |  |  |  |
| JMi-277/fSnorCo11 | | PEDV | | CHN | | 2015 | | KU363111.1 | |  |  |  |
| CH-SCYA-2-2014 | | PEDV | | CHN | | 2014 | | KU975423.1 | |  |  |  |
| CH-SCZG-2015 | | PEDV | | CHN | | 2015 | | KU975422.1 | |  |  |  |
| CH-SCYA-1-2014 | | PEDV | | CHN | | 2014 | | KU975420.1 | |  |  |  |
| CH-SCXC-2-2015 | | PEDV | | CHN | | 2015 | | KU975419.1 | |  |  |  |
| CH-SCXC-1-2015 | | PEDV | | CHN | | 2015 | | KU975418.1 | |  |  |  |
| CH-SCNC-3-2015 | | PEDV | | CHN | | 2015 | | KU975416.1 | |  |  |  |
| CH-SCNC-2-2015 | | PEDV | | CHN | | 2015 | | KU975415.1 | |  |  |  |
| CH-SCNC-1-2015 | | PEDV | | CHN | | 2015 | | KU975414.1 | |  |  |  |
| CH-SCMY-4-2014 | | PEDV | | CHN | | 2014 | | KU975413.1 | |  |  |  |
| CH-SCMY-3-2014 | | PEDV | | CHN | | 2014 | | KU975412.1 | |  |  |  |
| CH-SCMY-2-2014 | | PEDV | | CHN | | 2014 | | KU975411.1 | |  |  |  |
| CH-SCMY-1-2014 | | PEDV | | CHN | | 2014 | | KU975410.1 | |  |  |  |
| CH-SCJT-2015 | | PEDV | | CHN | | 2015 | | KU975409.1 | |  |  |  |
| CH-SCCD-2014 | | PEDV | | CHN | | 2014 | | KU975406.1 | |  |  |  |
| CH-SCAY-2014 | | PEDV | | CHN | | 2014 | | KU975405.1 | |  |  |  |
| CH-SCAY-2-2015 | | PEDV | | CHN | | 2015 | | KU975404.1 | |  |  |  |
| CH-SCAY-1-2015 | | PEDV | | CHN | | 2015 | | KU975403.1 | |  |  |  |
| CH-SCJY-2015 | | PEDV | | CHN | | 2015 | | KU975401.1 | |  |  |  |
| PEDV1-S-4 | | PEDV | | CHN | | 2015 | | KT313039.1 | |  |  |  |
| PEDV4-S-3 | | PEDV | | CHN | | 2015 | | KT313038.1 | |  |  |  |
| PEDV3-S-1 | | PEDV | | CHN | | 2015 | | KT313037.1 | |  |  |  |
| PEDV2-S-5 | | PEDV | | CHN | | 2015 | | KT313036.1 | |  |  |  |
| KPEDV-9 | | PEDV | | South Korea | | 2014 | | KF898124.1 | |  |  |  |
| GXNN/2013 | | PEDV | | CHN | | 2013 | | KF601201.1 | |  |  |  |
| GDEP/2013 | | PEDV | | CHN | | 2013 | | KF601200.1 | |  |  |  |
| GXHZ/2013 | | PEDV | | CHN | | 2013 | | KF601199.1 | |  |  |  |
| JXJA/2013 | | PEDV | | CHN | | 2013 | | KF601198.1 | |  |  |  |
| HNCZ/2013 | | PEDV | | CHN | | 2013 | | KF601197.1 | |  |  |  |
| JXGZ/2013 | | PEDV | | CHN | | 2013 | | KF601196.1 | |  |  |  |
| GXLZ/2013 | | PEDV | | CHN | | 2013 | | KF601195.1 | |  |  |  |
| MK | | PEDV | | Japan | | 2013 | | AB548624.1 | |  |  |  |
| NK | | PEDV | | Japan | | 2013 | | AB548623.1 | |  |  |  |
| KH | | PEDV | | Japan | | 2013 | | AB548622.1 | |  |  |  |
| 83P-5, 100th-passaged | | PEDV | | Japan | | 2013 | | AB548621.1 | |  |  |  |
| 83P-5, 61st-passaged | | PEDV | | Japan | | 2013 | | AB548620.1 | |  |  |  |
| 83P-5, 34th-passaged | | PEDV | | Japan | | 2013 | | AB548619.1 | |  |  |  |
| 83P-5 | | PEDV | | Japan | | 2013 | | AB548618.1 | |  |  |  |
| CH8 | | PEDV | | CHN | | 2012 | | JQ239436.1 | |  |  |  |
| CH7 | | PEDV | | CHN | | 2012 | | JQ239435.1 | |  |  |  |
| CH6 | | PEDV | | CHN | | 2012 | | JQ239434.1 | |  |  |  |
| CH5 | | PEDV | | CHN | | 2012 | | JQ239433.1 | |  |  |  |
| CH4 | | PEDV | | CHN | | 2012 | | JQ239432.1 | |  |  |  |
| CH3 | | PEDV | | CHN | | 2012 | | JQ239431.1 | |  |  |  |
| CH2 | | PEDV | | CHN | | 2012 | | JQ239430.1 | |  |  |  |
| CH1 | | PEDV | | CHN | | 2012 | | JQ239429.1 | |  |  |  |
| DR13 | | PEDV | | Korea | | 2007 | | DQ462404.2 | |  |  |  |
| CH/HNNY-01/2020 | | PEDV | | CHN | | 2020 | | MZ570149.1 | |  |  |  |
| CH/HNXC-04/2020 | | PEDV | | CHN | | 2020 | | MZ570148.1 | |  |  |  |
| CH/HNXC-03/2020 | | PEDV | | CHN | | 2020 | | MZ570147.1 | |  |  |  |
| CH/HNSQ-01/2020 | | PEDV | | CHN | | 2020 | | MZ570146.1 | |  |  |  |
| CH/HNKF-03/2020 | | PEDV | | CHN | | 2020 | | MZ570145.1 | |  |  |  |
| CH/HNAY-01/2020 | | PEDV | | CHN | | 2020 | | MZ570144.1 | |  |  |  |
| CH/HNSMX-01/2020 | | PEDV | | CHN | | 2020 | | MZ570143.1 | |  |  |  |
| CH/HNXC-02/2020 | | PEDV | | CHN | | 2020 | | MZ570142.1 | |  |  |  |
| CH/HNKF-02/2020 | | PEDV | | CHN | | 2020 | | MZ570141.1 | |  |  |  |
| CH/HNLH-02/2020 | | PEDV | | CHN | | 2020 | | MZ570140.1 | |  |  |  |
| CH/HNXC-01/2020 | | PEDV | | CHN | | 2020 | | MZ570139.1 | |  |  |  |
| CH/HNJZ-01/2020 | | PEDV | | CHN | | 2020 | | MZ570138.1 | |  |  |  |
| CH/HNXY-01/2020 | | PEDV | | CHN | | 2020 | | MZ570136.1 | |  |  |  |
| CH/HNLH-01/2020 | | PEDV | | CHN | | 2020 | | MZ570137.1 | |  |  |  |
| CH/HNXX-02/2020 | | PEDV | | CHN | | 2020 | | MZ570135.1 | |  |  |  |
| CH/HNXX-01/2020 | | PEDV | | CHN | | 2020 | | MZ570134.1 | |  |  |  |
| CH/HNKF-01/2020 | | PEDV | | CHN | | 2020 | | MZ570133.1 | |  |  |  |
| LNBX-2018 | | PEDV | | CHN | | 2018 | | MT294132.1 | |  |  |  |
| LNHD-2018 | | PEDV | | CHN | | 2018 | | MT294133.1 | |  |  |  |
| TJLT-2018 | | PEDV | | CHN | | 2018 | | MT294135.1 | |  |  |  |
| NMTL-2018 | | PEDV | | CHN | | 2018 | | MT294134.1 | |  |  |  |
| JSCF-2018 | | PEDV | | CHN | | 2018 | | MT294131.1 | |  |  |  |
| JLSD-2018 | | PEDV | | CHN | | 2018 | | MT294130.1 | |  |  |  |
| HLJYTX-2018 | | PEDV | | CHN | | 2018 | | MT294129.1 | |  |  |  |
| HBGJ-2018 | | PEDV | | CHN | | 2018 | | MT294128.1 | |  |  |  |
| GDsg13 | | PEDV | | CHN | | 2021 | | MW478772.1 | |  |  |  |
| GDsg12 | | PEDV | | CHN | | 2021 | | MW478771.1 | |  |  |  |
| GDsg11 | | PEDV | | CHN | | 2021 | | MW478770.1 | |  |  |  |
| GDsg10 | | PEDV | | CHN | | 2021 | | MW478769.1 | |  |  |  |
| GDsg09 | | PEDV | | CHN | | 2021 | | MW478768.1 | |  |  |  |
| GDsg08 | | PEDV | | CHN | | 2021 | | MW478767.1 | |  |  |  |
| GDsg07 | | PEDV | | CHN | | 2021 | | MW478766.1 | |  |  |  |
| GDsg06 | | PEDV | | CHN | | 2021 | | MW478765.1 | |  |  |  |
| GDsg04 | | PEDV | | CHN | | 2021 | | MW478763.1 | |  |  |  |
| GDsg05 | | PEDV | | CHN | | 2021 | | MW478764.1 | |  |  |  |
| GDsg03 | | PEDV | | CHN | | 2021 | | MW478762.1 | |  |  |  |
| GDsg02 | | PEDV | | CHN | | 2021 | | MW478761.1 | |  |  |  |
| GDsg01 | | PEDV | | CHN | | 2021 | | MW478760.1 | |  |  |  |
| SWUN-C6-CH-SCYA-2019 | | PEDV | | CHN | | 2019 | | MN161584.1 | |  |  |  |
| E16-39 | | PEDV | | Japan | | 2020 | | MK458305.1 | |  |  |  |
| E16-37 | | PEDV | | Japan | | 2020 | | MK458304.1 | |  |  |  |
| E16-32 | | PEDV | | Japan | | 2020 | | MK458303.1 | |  |  |  |
| E16-31 | | PEDV | | Japan | | 2020 | | MK458302.1 | |  |  |  |
| E14-74 | | PEDV | | Japan | | 2020 | | MK458301.1 | |  |  |  |
| E14-73 | | PEDV | | Japan | | 2020 | | MK458300.1 | |  |  |  |
| E14-71 | | PEDV | | Japan | | 2020 | | MK458299.1 | |  |  |  |
| E14-69 | | PEDV | | Japan | | 2020 | | MK458298.1 | |  |  |  |
| CT | | PEDV | | CHN | | 2020 | | MK539948.2 | |  |  |  |
| CH/SCQL-2/2018 | | PEDV | | CHN | | 2018 | | MN617867.1 | |  |  |  |
| CH/SCQL-1/2018 | | PEDV | | CHN | | 2018 | | MN617866.1 | |  |  |  |
| CH/SCYB-1/2018 | | PEDV | | CHN | | 2018 | | MN617865.1 | |  |  |  |
| CH/SCLS-1/2018 | | PEDV | | CHN | | 2018 | | MN617864.1 | |  |  |  |
| CH/SCGY-1/2018 | | PEDV | | CHN | | 2018 | | MN617863.1 | |  |  |  |
| CH/SCMY-1/2018 | | PEDV | | CHN | | 2018 | | MN617862.1 | |  |  |  |
| CH/SCZY-1/2018 | | PEDV | | CHN | | 2018 | | MN617861.1 | |  |  |  |
| CH/SCMS-1/2018 | | PEDV | | CHN | | 2018 | | MN617860.1 | |  |  |  |
| CH/SCCD-2/2018 | | PEDV | | CHN | | 2018 | | MN617859.1 | |  |  |  |
| CH/SCCD-1/2018 | | PEDV | | CHN | | 2018 | | MN617858.1 | |  |  |  |
| swun-H3-CH-SCYA-2019 | | PEDV | | CHN | | 2019 | | MK820042.1 | |  |  |  |
| swun-Y1-CH-SCCQ-2019 | | PEDV | | CHN | | 2019 | | MK820041.1 | |  |  |  |
| swun-H1-CH-SCYA-2019 | | PEDV | | CHN | | 2019 | | MK820040.1 | |  |  |  |
| swun-MY-CH-SCMY-2019 | | PEDV | | CHN | | 2019 | | MK820039.1 | |  |  |  |
| swun-3CH-CH-SCZG-2019 | | PEDV | | CHN | | 2019 | | MK820038.1 | |  |  |  |
| swun-18-CH-SCLS-2019 | | PEDV | | CHN | | 2019 | | MK820037.1 | |  |  |  |
| CH-SCNJ-2019 | | PEDV | | CHN | | 2019 | | MK685665.1 | |  |  |  |
| CH/SCXC/2018 | | PEDV | | CHN | | 2018 | | MK598821.1 | |  |  |  |
| CH/SCXC/2018 | | PEDV | | CHN | | 2018 | | MK598820.1 | |  |  |  |
| CH/SCZY/2018 | | PEDV | | CHN | | 2018 | | MK598819.1 | |  |  |  |
| SWUN2/CH/SCXC/2018 | | PEDV | | CHN | | 2018 | | MK592416.1 | |  |  |  |
| SWUN19/CH/SCZY/2018 | | PEDV | | CHN | | 2018 | | MK592415.1 | |  |  |  |
| KNU-1821 | | PEDV | | South Korea | | 2019 | | MK032695.1 | |  |  |  |
| KNU-1820 | | PEDV | | South Korea | | 2019 | | MK032694.1 | |  |  |  |
| KNU-1819 | | PEDV | | South Korea | | 2019 | | MK032693.1 | |  |  |  |
| KNU-1805 | | PEDV | | South Korea | | 2019 | | MH243317.1 | |  |  |  |
| KNU-1803 | | PEDV | | South Korea | | 2019 | | MH243315.1 | |  |  |  |
| KNU-1802 | | PEDV | | South Korea | | 2019 | | MH243314.1 | |  |  |  |
| KNU-1801 | | PEDV | | South Korea | | 2019 | | MH243313.1 | |  |  |  |
| CH/SCBC/2018 | | PEDV | | CHN | | 2018 | | MH593153.1 | |  |  |  |
| CH/SCJY710/2018 | | PEDV | | CHN | | 2018 | | MH678638.1 | |  |  |  |
| CH/SCXH/2018 | | PEDV | | CHN | | 2018 | | MH593151.1 | |  |  |  |
| CH/SCQL623/2018 | | PEDV | | CHN | | 2018 | | MH593152.1 | |  |  |  |
| CH/SCLS608/2018 | | PEDV | | CHN | | 2018 | | MH593150.1 | |  |  |  |
| CH/SCYA/2018 | | PEDV | | CHN | | 2018 | | MH593149.1 | |  |  |  |
| CH/SCCZ/2018 | | PEDV | | CHN | | 2018 | | MH593148.1 | |  |  |  |
| CH/SCPZ/2018 | | PEDV | | CHN | | 2018 | | MH593147.1 | |  |  |  |
| CH/SCZZ/2018 | | PEDV | | CHN | | 2018 | | MH593146.1 | |  |  |  |
| CH/SCSH525/2018 | | PEDV | | CHN | | 2018 | | MH593145.1 | |  |  |  |
| CH/SCDY523/2018 | | PEDV | | CHN | | 2018 | | MH593144.1 | |  |  |  |
| CH/SCDL/2018 | | PEDV | | CHN | | 2018 | | MH593143.1 | |  |  |  |
| CH/SCQL517/2018 | | PEDV | | CHN | | 2018 | | MH593142.1 | |  |  |  |
| CH/SCQS/2018 | | PEDV | | CHN | | 2018 | | MH593141.1 | |  |  |  |
| CH/SCDY503/2018 | | PEDV | | CHN | | 2018 | | MH593140.1 | |  |  |  |
| CH/SCSH425/2018 | | PEDV | | CHN | | 2018 | | MH593139.1 | |  |  |  |
| CH/GZJP/2017 | | PEDV | | CHN | | 2017 | | MH593138.1 | |  |  |  |
| CH/SCJY/2018 | | PEDV | | CHN | | 2018 | | MH053420.1 | |  |  |  |
| CH/SCCZ/2017 | | PEDV | | CHN | | 2017 | | MH053419.1 | |  |  |  |
| CH/SCAZ1/2017 | | PEDV | | CHN | | 2017 | | MH053418.1 | |  |  |  |
| CH/SCQX2/2017 | | PEDV | | CHN | | 2017 | | MH053417.1 | |  |  |  |
| CH/SCQX1/2017 | | PEDV | | CHN | | 2017 | | MH053416.1 | |  |  |  |
| CH/SCTJ/2017 | | PEDV | | CHN | | 2017 | | MH053415.1 | |  |  |  |
| CH/SCZJ/2017 | | PEDV | | CHN | | 2017 | | MH053414.1 | |  |  |  |
| CH/SCSH/2018 | | PEDV | | CHN | | 2018 | | MH053413.1 | |  |  |  |
| CH/SCMZ/2017 | | PEDV | | CHN | | 2017 | | MH053412.1 | |  |  |  |
| Unknow | | PEDV | | Korea | | 2018 | | MG602712.1 | |  |  |  |
| P5-V | | PEDV | | Japan | | 2018 | | KY619780.1 | |  |  |  |
| 96-P4C6 | | PEDV | | Japan | | 2018 | | KY619779.1 | |  |  |  |
| 16JM-339 | | PEDV | | Japan | | 2018 | | KY619778.1 | |  |  |  |
| 16JM-334 | | PEDV | | Japan | | 2018 | | KY619777.1 | |  |  |  |
| 16JM-326 | | PEDV | | Japan | | 2018 | | KY619776.1 | |  |  |  |
| 16JM-325 | | PEDV | | Japan | | 2018 | | KY619775.1 | |  |  |  |
| 16JM-323 | | PEDV | | Japan | | 2018 | | KY619774.1 | |  |  |  |
| 16JM-319 | | PEDV | | Japan | | 2018 | | KY619773.1 | |  |  |  |
| 15JM-315 | | PEDV | | Japan | | 2018 | | KY619772.1 | |  |  |  |
| 14JM-311 | | PEDV | | Japan | | 2018 | | KY619771.1 | |  |  |  |
| 14JM-297 | | PEDV | | Japan | | 2018 | | KY619770.1 | |  |  |  |
| 13JM-293 | | PEDV | | Japan | | 2018 | | KY619769.1 | |  |  |  |
| 13JM-291 | | PEDV | | Japan | | 2018 | | KY619768.1 | |  |  |  |
| 14JM-268 | | PEDV | | Japan | | 2018 | | KY619767.1 | |  |  |  |
| 14JM-248 | | PEDV | | Japan | | 2018 | | KY619766.1 | |  |  |  |
| 14JM-242 | | PEDV | | Japan | | 2018 | | KY619765.1 | |  |  |  |
| 14JM-236 | | PEDV | | Japan | | 2018 | | KY619764.1 | |  |  |  |
| 14JM-226 | | PEDV | | Japan | | 2018 | | KY619763.1 | |  |  |  |
| 14JM-216 | | PEDV | | Japan | | 2018 | | KY619762.1 | |  |  |  |
| 14JM-210 | | PEDV | | Japan | | 2018 | | KY619761.1 | |  |  |  |
| 14JM-208 | | PEDV | | Japan | | 2018 | | KY619760.1 | |  |  |  |
| 14JM-205 | | PEDV | | Japan | | 2018 | | KY619759.1 | |  |  |  |
| 14JM-200 | | PEDV | | Japan | | 2018 | | KY619758.1 | |  |  |  |
| 14JM-199 | | PEDV | | Japan | | 2018 | | KY619757.1 | |  |  |  |
| 14JM-181 | | PEDV | | Japan | | 2018 | | KY619756.1 | |  |  |  |
| 14JM-179 | | PEDV | | Japan | | 2018 | | KY619755.1 | |  |  |  |
| 14JM-168 | | PEDV | | Japan | | 2018 | | KY619754.1 | |  |  |  |
| 14JM-157 | | PEDV | | Japan | | 2018 | | KY619753.1 | |  |  |  |
| 14JM-152 | | PEDV | | Japan | | 2018 | | KY619752.1 | |  |  |  |
| 14JM-150 | | PEDV | | Japan | | 2018 | | KY619751.1 | |  |  |  |
| 14JM-147 | | PEDV | | Japan | | 2018 | | KY619750.1 | |  |  |  |
| 14JM-146 | | PEDV | | Japan | | 2018 | | KY619749.1 | |  |  |  |
| 14JM-144 | | PEDV | | Japan | | 2018 | | KY619748.1 | |  |  |  |
| 14JM-143 | | PEDV | | Japan | | 2018 | | KY619747.1 | |  |  |  |
| 14JM-142 | | PEDV | | Japan | | 2018 | | KY619746.1 | |  |  |  |
| 14JM-140 | | PEDV | | Japan | | 2018 | | KY619745.1 | |  |  |  |
| 14JM-139 | | PEDV | | Japan | | 2018 | | KY619744.1 | |  |  |  |
| 14JM-138 | | PEDV | | Japan | | 2018 | | KY619743.1 | |  |  |  |
| 13JM-128 | | PEDV | | Japan | | 2018 | | KY619742.1 | |  |  |  |
| 13JM-127 | | PEDV | | Japan | | 2018 | | KY619741.1 | |  |  |  |
| 14JM-126 | | PEDV | | Japan | | 2018 | | KY619740.1 | |  |  |  |
| 14JM-123 | | PEDV | | Japan | | 2018 | | KY619739.1 | |  |  |  |
| 14JM-119 | | PEDV | | Japan | | 2018 | | KY619738.1 | |  |  |  |
| 14JM-118 | | PEDV | | Japan | | 2018 | | KY619737.1 | |  |  |  |
| 14JM-40 | | PEDV | | Japan | | 2018 | | KY619736.1 | |  |  |  |
| 14JM-07 | | PEDV | | Japan | | 2018 | | KY619735.1 | |  |  |  |
| 14JM-01 | | PEDV | | Japan | | 2018 | | KY619734.1 | |  |  |  |
| PED-JS-2016-03 | | PEDV | | CHN | | 2016 | | MF038017.1 | |  |  |  |
| PED-JS-2015-12-1 | | PEDV | | CHN | | 2015 | | MF038016.1 | |  |  |  |
| PED-JS-2016-01 | | PEDV | | CHN | | 2016 | | MF038015.1 | |  |  |  |
| PED-JS-2016-07-2 | | PEDV | | CHN | | 2016 | | MF038014.1 | |  |  |  |
| PED-JS-2016-07-1 | | PEDV | | CHN | | 2016 | | MF038013.1 | |  |  |  |
| PED-JS-2016-05-5 | | PEDV | | CHN | | 2016 | | MF038012.1 | |  |  |  |
| PED-JS-2016-05-3 | | PEDV | | CHN | | 2016 | | MF038010.1 | |  |  |  |
| PED-JS-2016-05-4 | | PEDV | | CHN | | 2016 | | MF038011.1 | |  |  |  |
| PED-JS-2016-05-2 | | PEDV | | CHN | | 2016 | | MF038009.1 | |  |  |  |
| PED-JS-2016-05-1 | | | PEDV | | CHN | | 2016 | | MF038008.1 | |  |  |
| PED-JS-2015-12-4 | | | PEDV | | CHN | | 2015 | | MF038007.1 | |  |  |
| PED-JS-2016-12-1 | | | PEDV | | CHN | | 2016 | | MF038006.1 | |  |  |
| HBHG1 | | | PEDV | | CHN | | 2017 | | KY775045.1 | |  |  |
| HBXY5 | | | PEDV | | CHN | | 2017 | | KY775044.1 | |  |  |
| HBHG2 | | | PEDV | | CHN | | 2017 | | KY775046.1 | |  |  |
| HBHG3 | | | PEDV | | CHN | | 2017 | | KY775047.1 | |  |  |
| HBHG4 | | | PEDV | | CHN | | 2017 | | KY775048.1 | |  |  |
| HBHG6 | | | PEDV | | CHN | | 2017 | | KY775049.1 | |  |  |
| HBHG5 | | | PEDV | | CHN | | 2017 | | KY775050.1 | |  |  |
| HBEZ1 | | | PEDV | | CHN | | 2017 | | KY775051.1 | |  |  |
| HBEZ4 | | | PEDV | | CHN | | 2017 | | KY775052.1 | |  |  |
| HBEZ2 | | | PEDV | | CHN | | 2017 | | KY775053.1 | |  |  |
| HBEZ3 | | | PEDV | | CHN | | 2017 | | KY775054.1 | |  |  |
| HBYC1 | | | PEDV | | CHN | | 2017 | | KY775055.1 | |  |  |
| VN2-0514/SouthVietnam/2014 | | | PEDV | | Vietnam | | 2014 | | KR941552.1 | |  |  |
| VN1-0514/SouthVietnam/2014 | | | PEDV | | Vietnam | | 2014 | | KR941553.1 | |  |  |
| VN6-0514/SouthVietnam/2014 | | | PEDV | | Vietnam | | 2014 | | KR941554.1 | |  |  |
| VN0114-1/NorthVietnam/2013 | | | PEDV | | Vietnam | | 2013 | | KR941555.1 | |  |  |
| GF0413-1/NorthVietnam/2013 | | | PEDV | | Vietnam | | 2013 | | KR941556.1 | |  |  |
| PED-JS-2016-12-2 | | | PEDV | | CHN | | 2016 | | MF038005.1 | |  |  |
| PED-JS-2015-12-3 | | | PEDV | | CHN | | 2015 | | MF038004.1 | |  |  |
| PED-JS-2015-12-2 | | | PEDV | | CHN | | 2015 | | MF038003.1 | |  |  |
| HBXY4 | | | PEDV | | CHN | | 2017 | | KY775043.1 | |  |  |
| HBXY3 | | | PEDV | | CHN | | 2017 | | KY775042.1 | |  |  |
| HBXY2 | | | PEDV | | CHN | | 2017 | | KY775041.1 | |  |  |
| HBXY1 | | | PEDV | | CHN | | 2017 | | KY775040.1 | |  |  |
| HBJZ2 | | | PEDV | | CHN | | 2017 | | KY775039.1 | |  |  |
| BHJZ1 | | | PEDV | | CHN | | 2017 | | KY775038.1 | |  |  |
| HBJM3 | | | PEDV | | CHN | | 2017 | | KY775037.1 | |  |  |
| HBJM2 | | | PEDV | | CHN | | 2017 | | KY775036.1 | |  |  |
| HBJM1 | | | PEDV | | CHN | | 2017 | | KY775035.1 | |  |  |
| QIAP1401 | | PEDV | | Korea | | 2016 | | KX793713.1 | |  |  |  |
| YN4-144 | | PEDV | | CHN | | 2016 | | KM225252.1 | |  |  |  |
| YN4-120 | | PEDV | | CHN | | 2016 | | KM225251.1 | |  |  |  |
| YN4-90 | | PEDV | | CHN | | 2016 | | KM225250.1 | |  |  |  |
| YN4-60 | | PEDV | | CHN | | 2016 | | KM225249.1 | |  |  |  |
| YN4-30 | | PEDV | | CHN | | 2016 | | KM225248.1 | |  |  |  |
| YN4-15 | | PEDV | | CHN | | 2016 | | KM225247.1 | |  |  |  |
| YN4-9 | | PEDV | | CHN | | 2016 | | KM225246.1 | |  |  |  |
| YN4-1 | | PEDV | | CHN | | 2016 | | KM225245.1 | |  |  |  |
| HN40 | | PEDV | | CHN | | 2016 | | KM225244.1 | |  |  |  |
| HN27 | | PEDV | | CHN | | 2016 | | KM225243.1 | |  |  |  |
| HN26 | | PEDV | | CHN | | 2016 | | KM225242.1 | |  |  |  |
| HN41 | | PEDV | | CHN | | 2016 | | KM225241.1 | |  |  |  |
| FJ64 | | PEDV | | CHN | | 2016 | | KM225240.1 | |  |  |  |
| FJ63 | | PEDV | | CHN | | 2016 | | KM225239.1 | |  |  |  |
| CH-SDZC-2015 | | PEDV | | CHN | | 2015 | | KU133269.1 | |  |  |  |
| CH-SDZC-2012 | | PEDV | | CHN | | 2012 | | KU133268.1 | |  |  |  |
| CH-SDZB-2012 | | PEDV | | CHN | | 2012 | | KU133267.1 | |  |  |  |
| CH-SDYT-2012 | | PEDV | | CHN | | 2012 | | KU133266.1 | |  |  |  |
| CH-SDWF-2015 | | PEDV | | CHN | | 2015 | | KU133265.1 | |  |  |  |
| CH-SDWF-2012 | | PEDV | | CHN | | 2012 | | KU133264.1 | |  |  |  |
| CH-SDTA-2012 | | PEDV | | CHN | | 2012 | | KU133263.1 | |  |  |  |
| CH-SDRZ-2013 | | PEDV | | CHN | | 2013 | | KU133262.1 | |  |  |  |
| CH-SDQH-2014 | | PEDV | | CHN | | 2014 | | KU133261.1 | |  |  |  |
| CH-SDQF-5-2014 | | PEDV | | CHN | | 2014 | | KU133260.1 | |  |  |  |
| CH-SDQF-4-2014 | | PEDV | | CHN | | 2014 | | KU133259.1 | |  |  |  |
| CH-SDQF-3-2014 | | PEDV | | CHN | | 2014 | | KU133258.1 | |  |  |  |
| CH-SDQF-2-2014 | | PEDV | | CHN | | 2014 | | KU133257.1 | |  |  |  |
| CH-SDQF-1-2014 | | PEDV | | CHN | | 2014 | | KU133256.1 | |  |  |  |
| CH-SDLY-3-2014 | | PEDV | | CHN | | 2014 | | KU133255.1 | |  |  |  |
| CH-SDLY-2-2014 | | PEDV | | CHN | | 2014 | | KU133254.1 | |  |  |  |
| CH-SDLY-2-2013 | | PEDV | | CHN | | 2013 | | KU133253.1 | |  |  |  |
| CH-SDLY-2-2012 | | PEDV | | CHN | | 2012 | | KU133252.1 | |  |  |  |
| CH-SDLY-1-2014 | | PEDV | | CHN | | 2014 | | KU133251.1 | |  |  |  |
| CH-SDLY-1-2013 | | PEDV | | CHN | | 2013 | | KU133250.1 | |  |  |  |
| CH-SDLY-1-2012 | | PEDV | | CHN | | 2012 | | KU133249.1 | |  |  |  |
| CH-SDLS-2-2014 | | PEDV | | CHN | | 2014 | | KU133248.1 | |  |  |  |
| CH-SDLS-1-2014 | | PEDV | | CHN | | 2014 | | KU133247.1 | |  |  |  |
| CH-SDLQ-2015 | | PEDV | | CHN | | 2015 | | KU133246.1 | |  |  |  |
| CH-SDJY-2012 | | PEDV | | CHN | | 2012 | | KU133245.1 | |  |  |  |
| CH-SDHZ-4-2013 | | PEDV | | CHN | | 2013 | | KU133244.1 | |  |  |  |
| CH-SDHZ-3-2013 | | PEDV | | CHN | | 2013 | | KU133243.1 | |  |  |  |
| CH-SDHZ-2-2013 | | PEDV | | CHN | | 2013 | | KU133242.1 | |  |  |  |
| CH-SDHZ-1-2013 | | PEDV | | CHN | | 2013 | | KU133241.1 | |  |  |  |
| CH-SDDZ-2012 | | PEDV | | CHN | | 2012 | | KU133240.1 | |  |  |  |
| CH-SDDY-2012 | | PEDV | | CHN | | 2012 | | KU133239.1 | |  |  |  |
| CH-SDDP-3-2014 | | PEDV | | CHN | | 2014 | | KU133238.1 | |  |  |  |
| CH-SDDP-2-2014 | | PEDV | | CHN | | 2014 | | KU133237.1 | |  |  |  |
| CH-SDDP-1-2014 | | PEDV | | CHN | | 2014 | | KU133236.1 | |  |  |  |
| CH-SDCQ-2015 | | PEDV | | CHN | | 2015 | | KU133235.1 | |  |  |  |
| CH-SDCQ-2014 | | PEDV | | CHN | | 2014 | | KU133234.1 | |  |  |  |
| CH-SDBZ-2-2015 | | PEDV | | CHN | | 2015 | | KU133233.1 | |  |  |  |
| CH-SDBZ-1-2015 | | PEDV | | CHN | | 2015 | | KU133232.1 | |  |  |  |
| YC2014 | | PEDV | | CHN | | 2014 | | KT428879.1 | |  |  |  |
| CZ2014 | | PEDV | | CHN | | 2014 | | KT428878.1 | |  |  |  |
| ECUADOR/Cotopaxi/2014 | | PEDV | | ECUADOR | | 2014 | | KT336490.1 | |  |  |  |
| HN1303 | | PEDV | | CHN | | 2015 | | KR080551.1 | |  |  |  |
| CH/GD-09/2013 | | PEDV | | CHN | | 2013 | | KP870141.1 | |  |  |  |
| CH/GD-08/2013 | | PEDV | | CHN | | 2013 | | KP870140.1 | |  |  |  |
| CH/GD-29/2014 | | PEDV | | ECUADOR | | 2014 | | KP870139.1 | |  |  |  |
| CH/GD-28/2014 | | PEDV | | ECUADOR | | 2014 | | KP870138.1 | |  |  |  |
| CH/GD-27/2014 | | PEDV | | ECUADOR | | 2014 | | KP870137.1 | |  |  |  |
| CH/GD-26/2014 | | PEDV | | ECUADOR | | 2014 | | KP870136.1 | |  |  |  |
| CH/GD-25/2014 | | PEDV | | ECUADOR | | 2014 | | KP870135.1 | |  |  |  |
| CH/GD-24/2014 | | PEDV | | ECUADOR | | 2014 | | KP870134.1 | |  |  |  |
| CH/GD-23/2014 | | PEDV | | ECUADOR | | 2014 | | KP870133.1 | |  |  |  |
| CH/GD-22/2014 | | PEDV | | ECUADOR | | 2014 | | KP870132.1 | |  |  |  |
| CH/GD-21/2014 | | PEDV | | ECUADOR | | 2014 | | KP870131.1 | |  |  |  |
| CH/GD-20/2013 | | | | PEDV | | CHN | | 2013 | | KP870130.1 | |  |
| CH/GD-19/2013 | | | | PEDV | | CHN | | 2013 | | KP870129.1 | |  |
| CH/GD-18/2013 | | | | PEDV | | CHN | | 2013 | | KP870128.1 | |  |
| CH/GD-17/2013 | | | | PEDV | | CHN | | 2013 | | KP870127.1 | |  |
| CH/GD-16/2013 | | | | PEDV | | CHN | | 2013 | | KP870126.1 | |  |
| CH/GD-15/2013 | | | | PEDV | | CHN | | 2013 | | KP870125.1 | |  |
| CH/GD-14/2013 | | | | PEDV | | CHN | | 2013 | | KP870124.1 | |  |
| CH/GD-13/2013 | | | | PEDV | | CHN | | 2013 | | KP870123.1 | |  |
| CH/GD-12/2013 | | | | PEDV | | CHN | | 2013 | | KP870122.1 | |  |
| CH/GD-11/2013 | | | | PEDV | | CHN | | 2013 | | KP870121.1 | |  |
| CH/GD-10/2013 | | | | PEDV | | CHN | | 2013 | | KP870120.1 | |  |
| CH/GD-07/2012 | | | | PEDV | | CHN | | 2012 | | KP870119.1 | |  |
| CH/GD-06/2012 | | | | PEDV | | CHN | | 2012 | | KP870118.1 | |  |
| CH/GD-05/2012 | | | | PEDV | | CHN | | 2012 | | KP870117.1 | |  |
| CH/GD-04/2012 | | | | PEDV | | CHN | | 2012 | | KP870116.1 | |  |
| CH/GD-03/2012 | | | | PEDV | | CHN | | 2012 | | KP870115.1 | |  |
| CH/GD-02/2012 | | | | PEDV | | CHN | | 2012 | | KP870114.1 | |  |
| CH/GD-01/2012 | | | | PEDV | | CHN | | 2012 | | KP870113.1 | |  |
| CH/JSZL-N2/2013 | | | | PEDV | | CHN | | 2013 | | KF840562.1 | |  |
| CH/JSZL-S2/2013 | | | | PEDV | | CHN | | 2013 | | KF840561.1 | |  |
| CH/ZJQZ-2w/2012 | | | | PEDV | | CHN | | 2012 | | KF840557.1 | |  |
| GDS03 | | | | PEDV | | CHN | | 2014 | | AB857235.1 | |  |
| GDS02 | | | | PEDV | | CHN | | 2014 | | AB857234.1 | |  |
| GDS01 | | | | PEDV | | CHN | | 2014 | | AB857233.1 | |  |
| NJ | | | | PEDV | | CHN | | 2012 | | KJ642641.1 | |  |
| FJ-ZP 2014 | | | | PEDV | | CHN | | 2014 | | KJ646591.1 | |  |
| FJ-YX 2013 | | | | PEDV | | CHN | | 2013 | | KJ646590.1 | |  |
| FJ-QZ 2013 | | | | PEDV | | CHN | | 2013 | | KJ646589.1 | |  |
| FJ-QK 2013 | | | | PEDV | | CHN | | 2013 | | KJ646588.1 | |  |
| FJ-PT 2013 | | | | PEDV | | CHN | | 2013 | | KJ646587.1 | |  |
| FJ-ND 2013 | | | | PEDV | | CHN | | 2013 | | KJ646586.1 | |  |
| FJ-ND 2012 | | | | PEDV | | CHN | | 2012 | | KJ646585.1 | |  |
| FJ-LY 2013 | | | | PEDV | | CHN | | 2013 | | KJ646584.1 | |  |
| FJ-LY 2012 | | | | PEDV | | CHN | | 2012 | | KJ646583.1 | |  |
| FJ-FQ2 2012 | | | | PEDV | | CHN | | 2012 | | KJ646582.1 | |  |
| FJ-FQ1 2012 | | | | PEDV | | CHN | | 2012 | | KJ646581.1 | |  |
| FJ-FQ 2014 | | | | PEDV | | CHN | | 2014 | | KJ646580.1 | |  |
| FJ-CL 2013 | | | | PEDV | | CHN | | 2013 | | KJ646579.1 | |  |
| FJ-ZP 2013 | | | | PEDV | | CHN | | 2013 | | KJ646578.1 | |  |
| GDZQ/2012 | | | | PEDV | | CHN | | 2012 | | KF546804.1 | |  |
| GDJM/2012 | | | | PEDV | | CHN | | 2012 | | KF546803.1 | |  |
| GDDG/2011 | | | | PEDV | | CHN | | 2011 | | KF546802.1 | |  |
| GDQY/2011 | | | | PEDV | | CHN | | 2011 | | KF546801.1 | |  |
| GDFS/2011 | | | | PEDV | | CHN | | 2011 | | KF546800.1 | |  |
| XS2013 | | | | PEDV | | USA | | 2013 | | KF468755.1 | |  |
| GJL | | | | PEDV | | CHN | | 2013 | | KF294256.1 | |  |
| HZXS-2 | | | | PEDV | | CHN | | 2013 | | KF294255.1 | |  |
| HZXS-1 | | | | PEDV | | CHN | | 2013 | | KF294254.1 | |  |
| YJ7C | | | | PEDV | | CHN | | 2013 | | KF177258.1 | |  |
| YJ3F | | | | PEDV | | CHN | | 2013 | | KF177257.1 | |  |
| JY7C | | | | PEDV | | CHN | | 2013 | | KF177256.1 | |  |
| JY6C | | | | PEDV | | CHN | | 2013 | | KF177255.1 | |  |
| JY5C | | | | PEDV | | CHN | | 2013 | | KF177254.1 | |  |
| AD03 | | | | PEDV | | Korea | | 2013 | | KC879282.1 | |  |
| AD02 | | | | PEDV | | Korea | | 2013 | | KC879281.1 | |  |
| AD01 | | | | PEDV | | Korea | | 2013 | | KC879280.1 | |  |
| NJ02 | | | | PEDV | | Korea | | 2013 | | KC879279.1 | |  |
| NJ01 | | | | PEDV | | Korea | | 2013 | | KC879278.1 | |  |
| AS03 | | | | PEDV | | Korea | | 2013 | | KC879277.1 | |  |
| AS02 | | | | PEDV | | Korea | | 2013 | | KC879276.1 | |  |
| AS01 | | | | PEDV | | Korea | | 2013 | | KC879275.1 | |  |
| CH22-JS | | | | PEDV | | CHN | | 2012 | | JQ979290.1 | |  |
| CH17-GZ | | | | PEDV | | CHN | | 2012 | | JQ979289.1 | |  |
| CH13-GX | | | | PEDV | | CHN | | 2012 | | JQ979288.1 | |  |
| CHGD-01 | | | | PEDV | | CHN | | 2012 | | JN980698.1 | |  |
| CH9-FJ | | | | PEDV | | CHN | | 2012 | | JQ979287.1 | |  |
| CV777 | | | | PEDV | | CHN | | 2012 | | JN599150.1 | |  |
| CH/JLGZL/2011 | | | | PEDV | | CHN | | 2011 | | JQ638923.1 | |  |
| CH/JL/2011 | | | | PEDV | | CHN | | 2011 | | JQ638924.1 | |  |
| CH/HBQHD/2011 | | | | PEDV | | CHN | | 2011 | | JQ638922.1 | |  |
| CH/BJSY/2011 | | | | PEDV | | CHN | | 2011 | | JQ638921.1 | |  |
| CH/JLCC/2011 | | | | PEDV | | CHN | | 2011 | | JQ638920.1 | |  |
| CH/SDQD/2011 | | | | PEDV | | CHN | | 2011 | | JQ638919.1 | |  |
| CH/HBBD/2011 | | | | PEDV | | CHN | | 2011 | | JQ638918.1 | |  |
| CH/SDLY/2011 | | | | PEDV | | CHN | | 2011 | | JQ638917.1 | |  |
| CH/HLJHH/2011 | | | | PEDV | | CHN | | 2011 | | JQ638916.1 | |  |
| CH/GD/2011 | | | | PEDV | | CHN | | 2011 | | JQ638915.1 | |  |
| CH/FJND-1/2011 | | | | PEDV | | CHN | | 2011 | | JN543367.1 | |  |
| CH/FJND-3/2011 | | | | PEDV | | CHN | | 2011 | | JN381492.1 | |  |
| CH/FJND-2/2011 | | | | PEDV | | CHN | | 2011 | | JN315706.1 | |  |
| JS-2004-2 | | | | PEDV | | CHN | | 2004 | | Y653204.1 | |  |
| SDHY_BZ | | | | PEDV | | CHN | | 2022 | | ON988096.1 | |  |
| SDHY_JN01 | | | | PEDV | | CHN | | 2022 | | ON988095.1 | |  |
| SDHY_JN02 | | | | PEDV | | CHN | | 2022 | | ON988094.1 | |  |
| SDHY_LC | | | | PEDV | | CHN | | 2022 | | ON988093.1 | |  |
| SDHY_LW | | | | PEDV | | CHN | | 2022 | | ON988092.1 | |  |
| SDHY_QD | | | | PEDV | | CHN | | 2022 | | ON988091.1 | |  |
| SDHY_TA01 | | | | PEDV | | CHN | | 2022 | | ON988090.1 | |  |
| SDHY_TA02 | | | | PEDV | | CHN | | 2022 | | ON988089.1 | |  |
| SDHY_TA03 | | | | PEDV | | CHN | | 2022 | | ON988088.1 | |  |
| SDHY_YT | | | | PEDV | | CHN | | 2022 | | ON988087.1 | |  |
| SDHY_ZB | | | | PEDV | | CHN | | 2022 | | ON988086.1 | |  |
| SDHY_DY | | | | PEDV | | CHN | | 2022 | | ON988085.1 | |  |
| HT/2017 P150 | | | | PEDV | | CHN | | 2017 | | MW915437.1 | |  |
| HT/2017 P120 | | | | PEDV | | CHN | | 2017 | | MW915436.1 | |  |
| TQ/2017 P120 | | | | PEDV | | CHN | | 2017 | | MW915435.1 | |  |
| HY/2017 P150 | | | | PEDV | | CHN | | 2017 | | MW915434.1 | |  |
| HH/2017 P90 | | | | PEDV | | CHN | | 2017 | | MW915433.1 | |  |
| YJH/2015 P170 | | | | PEDV | | CHN | | 2015 | | MW915432.1 | |  |
| YJH/2015 P140 | | | | PEDV | | CHN | | 2015 | | MW915431.1 | |  |
| YJH/2015 P110 | | | | PEDV | | CHN | | 2015 | | MW915430.1 | |  |
| YJH/2015 P90 | | | | PEDV | | CHN | | 2015 | | MW915429.1 | |  |
| YJH/2015 | | | | PEDV | | CHN | | 2015 | | MW915428.1 | |  |
| HG/2017 P150 | | | | PEDV | | CHN | | 2017 | | MW915427.1 | |  |
| HG/2017 P120 | | | | PEDV | | CHN | | 2017 | | MW915426.1 | |  |
| HG/2017 P90 | | | | PEDV | | CHN | | 2017 | | MW915425.1 | |  |
| HG/2017 P30 | | | | PEDV | | CHN | | 2017 | | MW915424.1 | |  |
| SX/2017 P150 | | | | PEDV | | CHN | | 2017 | | MW915423.1 | |  |
| SX/2017 P90 | | | | PEDV | | CHN | | 2017 | | MW915422.1 | |  |
| SX/2017 P60 | | | | PEDV | | CHN | | 2017 | | MW915421.1 | |  |
| MT/2017 P90 | | | | PEDV | | CHN | | 2017 | | MW915420.1 | |  |
| FH/2017 | | | | PEDV | | CHN | | 2017 | | MW915419.1 | |  |
| CY/2017 P120 | | | | PEDV | | CHN | | 2017 | | MW915418.1 | |  |
| ZJC/2017 | | | | PEDV | | CHN | | 2017 | | MW826599.1 | |  |
| XT/2017 | | | | PEDV | | CHN | | 2017 | | MW826598.1 | |  |
| WF/2017 | | | | PEDV | | CHN | | 2017 | | MW826597.1 | |  |
| TQ/2017 | | | | PEDV | | CHN | | 2017 | | MW826596.1 | |  |
| LP/2017 | | | | PEDV | | CHN | | 2017 | | MW826595.1 | |  |
| HH/2017 | | | | PEDV | | CHN | | 2017 | | MW826594.1 | |  |
| FY/2017 | | | | PEDV | | CHN | | 2017 | | MW826593.1 | |  |
| CH/SCDZ-1/2020 | | | | PEDV | | CHN | | 2020 | | MW452940.1 | |  |
| SCGYSWUN02 | | | | PEDV | | CHN | | 2022 | | OL944723.1 | |  |
| CH/SCNJ-1/2020 | | | | PEDV | | CHN | | 2020 | | MW145535.1 | |  |
| CH/SCMS-2/2020 | | | | PEDV | | CHN | | 2020 | | MW145534.1 | |  |
| CH/SCZY-2/2020 | | | | PEDV | | CHN | | 2020 | | MW145533.1 | |  |
| CH/SCDY-1/2020 | | | | PEDV | | CHN | | 2020 | | MW145532.1 | |  |
| CH/SCYB-2/2019 | | | | PEDV | | CHN | | 2019 | | MW145531.1 | |  |
| CH/SCMY-2/2019 | | | | PEDV | | CHN | | 2019 | | MW145530.1 | |  |
| KNU-1912 | | | | PEDV | | South Korea | | 2022 | | MW560730.1 | |  |
| KNU-1911 | | | | PEDV | | South Korea | | 2022 | | MW560729.1 | |  |
| KNU-1907-11 | | | | PEDV | | South Korea | | 2022 | | MW560728.1 | |  |
| KNU-1907-10 | | | | PEDV | | South Korea | | 2022 | | MW560727.1 | |  |
| KNU-1907-9 | | | | PEDV | | South Korea | | 2022 | | MW560726.1 | |  |
| KNU-1907-7 | | | | PEDV | | South Korea | | 2022 | | MW560725.1 | |  |
| KNU-1907-6 | | | | PEDV | | South Korea | | 2022 | | MW560724.1 | |  |
| KNU-1907-5 | | | | PEDV | | South Korea | | 2022 | | MW560723.1 | |  |
| KNU-1907-4 | | | | PEDV | | South Korea | | 2022 | | MW560722.1 | |  |
| KNU-1907-2 | | | | PEDV | | South Korea | | 2022 | | MW560721.1 | |  |
| KNU-1907-1 | | | | PEDV | | South Korea | | 2022 | | MW560720.1 | |  |
| KNU-1908 | | | | PEDV | | South Korea | | 2022 | | MW560719.1 | |  |
| KNU-1836 | | | | PEDV | | South Korea | | 2022 | | MW560718.1 | |  |
| CH/SDJN/07/2020 | | | | PEDV | | CHN | | 2020 | | MZ161089.1 | |  |
| CH/SCLZ/12/2020 | | | | PEDV | | CHN | | 2020 | | MZ161088.1 | |  |
| CH/NMGTL/04/2020 | | | | PEDV | | CHN | | 2020 | | MZ161087.1 | |  |
| CH/GXLB/05/2020 | | | | PEDV | | CHN | | 2020 | | MZ161086.1 | |  |
| CH/SDLQ/09/2020 | | | | PEDV | | CHN | | 2020 | | MZ161085.1 | |  |
| CH/JSXZLD/10/2020 | | | | PEDV | | CHN | | 2020 | | MZ161084.1 | |  |
| CH/HBTS/09/2020 | | | | PEDV | | CHN | | 2020 | | MZ161083.1 | |  |
| CH/JSHY/09/2020 | | | | PEDV | | CHN | | 2020 | | MZ161082.1 | |  |
| CH/SCST/04/2020 | | | | PEDV | | CHN | | 2020 | | MZ161081.1 | |  |
| CH/GZSB/05/2020 | | | | PEDV | | CHN | | 2020 | | MZ161080.1 | |  |
| CH/SCDY/05/2020 | | | | PEDV | | CHN | | 2020 | | MZ161079.1 | |  |
| CH/SCYT/05/2020 | | | | PEDV | | CHN | | 2020 | | MZ161078.1 | |  |
| CH/SDLY/12/2020 | | | | PEDV | | CHN | | 2020 | | MZ161077.1 | |  |
| CH/SCGA/03/2021 | | | | PEDV | | CHN | | 2021 | | MZ161076.1 | |  |
| CH/GDYJ/03/2021 | | | | PEDV | | CHN | | 2021 | | MZ161075.1 | |  |
| CH/HNSQ/02/2021 | | | | PEDV | | CHN | | 2021 | | MZ161074.1 | |  |
| CH/HBSY/02/2021 | | | | PEDV | | CHN | | 2021 | | MZ161073.1 | |  |
| CH/JSLYG/02/2021 | | | | PEDV | | CHN | | 2021 | | MZ161072.1 | |  |
| CH/HBHA/01/2021 | | | | PEDV | | CHN | | 2021 | | MZ161071.1 | |  |
| CH/JXXG-2/01/2021 | | | | PEDV | | CHN | | 2021 | | MZ161070.1 | |  |
| CH/JXXG/01/2021 | | | | PEDV | | CHN | | 2021 | | MZ161069.1 | |  |
| CH/JSXZ/01/2021 | | | | PEDV | | CHN | | 2021 | | MZ161068.1 | |  |
| CH/HNBR/01/2021 | | | | PEDV | | CHN | | 2021 | | MZ161067.1 | |  |
| CH/HBXY/12/2020 | | | | PEDV | | CHN | | 2020 | | MZ161066.1 | |  |
| CH/HNLX/12/2020 | | | | PEDV | | CHN | | 2020 | | MZ161065.1 | |  |
| CH/JXPY/12/2020 | | | | PEDV | | CHN | | 2020 | | MZ161064.1 | |  |
| CH/GZZY/12/2020 | | | | PEDV | | CHN | | 2020 | | MZ161063.1 | |  |
| CH/AHXC/12/2020 | | | | PEDV | | CHN | | 2020 | | MZ161062.1 | |  |
| CH/HBZJC/11/2020 | | | | PEDV | | CHN | | 2020 | | MZ161061.1 | |  |
| CH/GXLP/07/2020 | | | | PEDV | | CHN | | 2020 | | MZ161060.1 | |  |
| CH/HNLY/03/2020 | | | | PEDV | | CHN | | 2020 | | MZ161059.1 | |  |
| CH/SCJS/03/2020 | | | | PEDV | | CHN | | 2020 | | MZ161058.1 | |  |
| CH/HBZL/03/2020 | | | | PEDV | | CHN | | 2020 | | MZ161057.1 | |  |
| CH/HNCC/03/2020 | | | | PEDV | | CHN | | 2020 | | MZ161056.1 | |  |
| CH/JXGZ/04/2020 | | | | PEDV | | CHN | | 2020 | | MZ161055.1 | |  |
| CH/FJDH/04/2020 s | | | | PEDV | | CHN | | 2020 | | MZ161054.1 | |  |
| CH/GDCY/04/2020 | | | | PEDV | | CHN | | 2020 | | MZ161053.1 | |  |
| CH/JXJJ/04/2020 | | | | PEDV | | CHN | | 2020 | | MZ161052.1 | |  |
| CH/GXLB/04/2020 | | | | PEDV | | CHN | | 2020 | | MZ161051.1 | |  |
| CH/GXNN/04/2020 | | | | PEDV | | CHN | | 2020 | | MZ161050.1 | |  |
| CH/SCPL/05/2020 | | | | PEDV | | CHN | | 2020 | | MZ161049.1 | |  |
| CH/LNPJ/05/2020 | | | | PEDV | | CHN | | 2020 | | MZ161048.1 | |  |
| CH/HNZZ/06/2020 | | | | PEDV | | CHN | | 2020 | | MZ161047.1 | |  |
| CH/SCMY/06/2020 | | | | PEDV | | CHN | | 2020 | | MZ161046.1 | |  |
| CH/GDCY/06/2020 | | | | PEDV | | CHN | | 2020 | | MZ161045.1 | |  |
| CH/AHMJ/07/2020 | | | | PEDV | | CHN | | 2020 | | MZ161044.1 | |  |
| CH/HBCZ/07/2020 | | | | PEDV | | CHN | | 2020 | | MZ161043.1 | |  |
| CH/SDZH/07/2020 | | | | PEDV | | CHN | | 2020 | | MZ161042.1 | |  |
| CH/JXPY/07/2020 | | | | PEDV | | CHN | | 2020 | | MZ161041.1 | |  |
| CH/GXLA/07/2020 | | | | PEDV | | CHN | | 2020 | | MZ161040.1 | |  |
| CH/LNFS/09/2020 | | | | PEDV | | CHN | | 2020 | | MZ161039.1 | |  |
| CH/JXHY/09/2020 | | | | PEDV | | CHN | | 2020 | | MZ161038.1 | |  |
| CH/SCCN/08/2020 | | | | PEDV | | CHN | | 2020 | | MZ161037.1 | |  |
| CH/SCLZ/09/2020 | | | | PEDV | | CHN | | 2020 | | MZ161036.1 | |  |
| CH/AHLA/10/2020 | | | | PEDV | | CHN | | 2020 | | MZ161035.1 | |  |
| CH/SCPZ/10/2020 | | | | PEDV | | CHN | | 2020 | | MZ161034.1 | |  |
| CH/JSJH/10/2020 | | | | PEDV | | CHN | | 2020 | | MZ161033.1 | |  |
| CH/JSSQ/10/2020 | | | | PEDV | | CHN | | 2020 | | MZ161032.1 | |  |
| CH/HBTS/10/2020 | | | | PEDV | | CHN | | 2020 | | MZ161031.1 | |  |
| CH/AHBZ/10/2020 | | | | PEDV | | CHN | | 2020 | | MZ161030.1 | |  |
| CH/HBHS/10/2020 | | | | PEDV | | CHN | | 2020 | | MZ161029.1 | |  |
| CH/HBHG/10/2020 | | | | PEDV | | CHN | | 2020 | | MZ161028.1 | |  |
| CH/GXLZ/10/2020 | | | | PEDV | | CHN | | 2020 | | MZ161027.1 | |  |
| CH/HBMZ/10/2020 | | | | PEDV | | CHN | | 2020 | | MZ161026.1 | |  |
| CH/HBSZ/10/2020 | | | | PEDV | | CHN | | 2020 | | MZ161025.1 | |  |
| CH/HBBX/10/2020 | | | | PEDV | | CHN | | 2020 | | MZ161024.1 | |  |
| CH/GXDX/10/2020 | | | | PEDV | | CHN | | 2020 | | MZ161023.1 | |  |
| CH/HNXX/11/2020 | | | | PEDV | | CHN | | 2020 | | MZ161022.1 | |  |
| CH/JXGZ/11/2020 | | | | PEDV | | CHN | | 2020 | | MZ161021.1 | |  |
| CH/SDNW-2/11/2020 | | | | PEDV | | CHN | | 2020 | | MZ161020.1 | |  |
| CH/YNDL/11/2020 | | | | PEDV | | CHN | | 2020 | | MZ161019.1 | |  |
| CH/HBXT/11/2020 | | | | PEDV | | CHN | | 2020 | | MZ161018.1 | |  |
| CH/SXXY/11/2020 | | | | PEDV | | CHN | | 2020 | | MZ161017.1 | |  |
| CH/JXXG/11/2020 | | | | PEDV | | CHN | | 2020 | | MZ161016.1 | |  |
| CH/SDNW/11/2020 | | | | PEDV | | CHN | | 2020 | | MZ161015.1 | |  |
| CH/GDYD/12/2020 | | | | PEDV | | CHN | | 2020 | | MZ161014.1 | |  |
| CH/AHBB/12/2020 | | | | PEDV | | CHN | | 2020 | | MZ161013.1 | |  |
| CH/SCMY/12/2020 | | | | PEDV | | CHN | | 2020 | | MZ161012.1 | |  |
| CH/HNCD/12/2020 | | | | PEDV | | CHN | | 2020 | | MZ161011.1 | |  |
| CH/JSXZGS/12/2020 | | | | PEDV | | CHN | | 2020 | | MZ161010.1 | |  |
| CH/SCCD/12/2020 | | | | PEDV | | CHN | | 2020 | | MZ161009.1 | |  |
| CH/GDMM/12/2020 | | | | PEDV | | CHN | | 2020 | | MZ161008.1 | |  |
| CH/HBSY/12/2020 | | | | PEDV | | CHN | | 2020 | | MZ161007.1 | |  |
| CH/JSSZ/12/2020 | | | | PEDV | | CHN | | 2020 | | MZ161006.1 | |  |
| CH/JSHA/12/2020 | | | | PEDV | | CHN | | 2020 | | MZ161005.1 | |  |
| CH/GXGG/12/2020 | | | | PEDV | | CHN | | 2020 | | MZ161004.1 | |  |
| CH/SCYBZH/12/2020 | | | | PEDV | | CHN | | 2020 | | MZ161003.1 | |  |
| CH/GDSG/12/2020 | | | | PEDV | | CHN | | 2020 | | MZ161002.1 | |  |
| CH/GDHG/12/2020 | | | | PEDV | | CHN | | 2020 | | MZ161001.1 | |  |
| CH/SCYB/12/2020 | | | | PEDV | | CHN | | 2020 | | MZ161000.1 | |  |
| CH/JSXZ/12/2020 | | | | PEDV | | CHN | | 2020 | | MZ160999.1 | |  |
| POR-VC102 s | | | | PEDV | | Spain | | 2021 | | MW251378.1 | |  |
| SP-VC101 | | | | PEDV | | Spain | | 2021 | | MW251377.1 | |  |
| SP-VC99 | | | | PEDV | | Spain | | 2021 | | MW251376.1 | |  |
| SP-VC98 | | | | PEDV | | Spain | | 2021 | | MW251375.1 | |  |
| SP-VC97 | | | | PEDV | | Spain | | 2021 | | MW251374.1 | |  |
| SP-VC96 | | | | PEDV | | Spain | | 2021 | | MW251373.1 | |  |
| SP-VC95 | | | | PEDV | | Spain | | 2021 | | MW251372.1 | |  |
| SP-VC94 | | | | PEDV | | Spain | | 2021 | | MW251371.1 | |  |
| SP-VC93 | | | | PEDV | | Spain | | 2021 | | MW251370.1 | |  |
| SP-VC92 | | | | PEDV | | Spain | | 2021 | | MW251369.1 | |  |
| SP-VC90 | | | | PEDV | | Spain | | 2021 | | MW251368.1 | |  |
| SP-VC89 | | | | PEDV | | Spain | | 2021 | | MW251367.1 | |  |
| SP-VT108 | | | | PEDV | | Spain | | 2021 | | MW251366.1 | |  |
| SP-VT87 | | | | PEDV | | Spain | | 2021 | | MW251365.1 | |  |
| SP-VT86 | | | | PEDV | | Spain | | 2021 | | MW251364.1 | |  |
| SP-VC87 | | | | PEDV | | Spain | | 2021 | | MW251363.1 | |  |
| SP-VC81 | | | | PEDV | | Spain | | 2021 | | MW251362.1 | |  |
| SP-VC77 | | | | PEDV | | Spain | | 2021 | | MW251361.1 | |  |
| SP-VC75 | | | | PEDV | | Spain | | 2021 | | MW251360.1 | |  |
| SP-VC68 | | | | PEDV | | Spain | | 2021 | | MW251359.1 | |  |
| SP-VC66 | | | | PEDV | | Spain | | 2021 | | MW251358.1 | |  |
| SP-VC63 | | | | PEDV | | Spain | | 2021 | | MW251357.1 | |  |
| SP-VC61 | | | | PEDV | | Spain | | 2021 | | MW251356.1 | |  |
| SP-VC55 | | | | PEDV | | Spain | | 2021 | | MW251355.1 | |  |
| SP-VC54 | | | | PEDV | | Spain | | 2021 | | MW251354.1 | |  |
| SP-VT13 | | | | PEDV | | Spain | | 2021 | | MW251353.1 | |  |
| SP-VC53 | | | | PEDV | | Spain | | 2021 | | MW251352.1 | |  |
| SP-VC52 | | | | PEDV | | Spain | | 2021 | | MW251351.1 | |  |
| SP-VC46 | | | | PEDV | | Spain | | 2021 | | MW251350.1 | |  |
| SP-VC29 | | | | PEDV | | Spain | | 2021 | | MW251349.1 | |  |
| SP-VC27 | | | | PEDV | | Spain | | 2021 | | MW251348.1 | |  |
| SP-VC19 | | | | PEDV | | Spain | | 2021 | | MW251347.1 | |  |
| SP-VC18 | | | | PEDV | | Spain | | 2021 | | MW251346.1 | |  |
| SP-VC16 | | | | PEDV | | Spain | | 2021 | | MW251345.1 | |  |
| SP-VC4 | | | | PEDV | | Spain | | 2021 | | MW251344.1 | |  |
| SP-VC3 | | | | PEDV | | Spain | | 2021 | | MW251343.1 | |  |
| CH/CC-2/2018 | | | | PEDV | | CHN | | 2018 | | MT031820.1 | |  |
| CH/CC-1/2018 | | | | PEDV | | CHN | | 2018 | | MT031819.1 | |  |
| CH/QD/2018 | | | | PEDV | | CHN | | 2018 | | MT031818.1 | |  |
| 1613-Murcia-Fuentealamo | | | | PEDV | | Spain | | 2020 | | MN692769.1 | |  |
| 1611-Murcia-Lorca | | | | PEDV | | Spain | | 2020 | | MN692768.1 | |  |
| 1587-7-Lugo-Chantada | | | | PEDV | | Spain | | 2020 | | MN692767.1 | |  |
| 1587-1-Ourense-Almoite | | | | PEDV | | Spain | | 2020 | | MN692766.1 | |  |
| 1576-Zamora-Santibanez | | | | PEDV | | Spain | | 2020 | | MN692765.1 | |  |
| 1573-Toledo-Pueblanueva | | | | PEDV | | Spain | | 2020 | | MN692764.1 | |  |
| 1556-Valencia-Requena | | | | PEDV | | Spain | | 2020 | | MN692763.1 | |  |
| GDzj18-2 | | | | PEDV | | CHN | | 2020 | | MN368725.1 | |  |
| GDzj18-1 | | | | PEDV | | CHN | | 2020 | | MN368724.1 | |  |
| GDst18 | | | | PEDV | | CHN | | 2020 | | MN368723.1 | |  |
| GDsg18-2 | | | | PEDV | | CHN | | 2020 | | MN368722.1 | |  |
| GDsg18-1 | | | | PEDV | | CHN | | 2020 | | MN368721.1 | |  |
| GDmm18-2 | | | | PEDV | | CHN | | 2020 | | MN368720.1 | |  |
| GDmm18-1 | | | | PEDV | | CHN | | 2020 | | MN368719.1 | |  |
| GDjm18-2 | | | | PEDV | | CHN | | 2020 | | MN368718.1 | |  |
| GDjm18-1 | | | | PEDV | | CHN | | 2020 | | MN368717.1 | |  |
| GDhz18 | | | | PEDV | | CHN | | 2020 | | MN368716.1 | |  |
| GDhy18-3 | | | | PEDV | | CHN | | 2020 | | MN368715.1 | |  |
| GDhy18-2 | | | | PEDV | | CHN | | 2020 | | MN368714.1 | |  |
| GDhy18-1 | | | | PEDV | | CHN | | 2020 | | MN368713.1 | |  |
| FJqz18 | | | | PEDV | | CHN | | 2020 | | MN368712.1 | |  |
| FJfz18-2 | | | | PEDV | | CHN | | 2020 | | MN368711.1 | |  |
| FJfz18-1 | | | | PEDV | | CHN | | 2020 | | MN368710.1 | |  |
| SDbz18 | | | | PEDV | | CHN | | 2020 | | MN368709.1 | |  |
| JXnc17 | | | | PEDV | | CHN | | 2020 | | MN368708.1 | |  |
| HNcz17 | | | | PEDV | | CHN | | 2020 | | MN368707.1 | |  |
| GDsg17 | | | | PEDV | | CHN | | 2020 | | MN368706.1 | |  |
| GDmm17-2 | | | | PEDV | | CHN | | 2020 | | MN368705.1 | |  |
| GDmm17-1 | | | | PEDV | | CHN | | 2020 | | MN368704.1 | |  |
| GDjm17-3 | | | | PEDV | | CHN | | 2020 | | MN368703.1 | |  |
| GDjm17-2 | | | | PEDV | | CHN | | 2020 | | MN368702.1 | |  |
| GDjm17-1 | | | | PEDV | | CHN | | 2020 | | MN368701.1 | |  |
| GDhz17 | | | | PEDV | | CHN | | 2020 | | MN368700.1 | |  |
| GDhy17 | | | | PEDV | | CHN | | 2020 | | MN368699.1 | |  |
| FJqz17-2 | | | | PEDV | | CHN | | 2020 | | MN368698.1 | |  |
| FJqz17-1 | | | | PEDV | | CHN | | 2020 | | MN368697.1 | |  |
| FJfz17-2 | | | | PEDV | | CHN | | 2020 | | MN368696.1 | |  |
| FJfz17-1 | | | | PEDV | | CHN | | 2020 | | MN368695.1 | |  |
| SCmy17 | | | | PEDV | | CHN | | 2020 | | MN368694.1 | |  |
| GDdg17 | | | | PEDV | | CHN | | 2020 | | MN368693.1 | |  |
| GDyj16 | | | | PEDV | | CHN | | 2020 | | MN368692.1 | |  |
| GDsg16-2 | | | | PEDV | | CHN | | 2020 | | MN368691.1 | |  |
| GDsg16-1 | | | | PEDV | | CHN | | 2020 | | MN368690.1 | |  |
| GDjx16 | | | | PEDV | | CHN | | 2020 | | MN368689.1 | |  |
| GDjm16-1 | | | | PEDV | | CHN | | 2020 | | MN368687.1 | |  |
| GDjm16-2 | | | | PEDV | | CHN | | 2020 | | MN368688.1 | |  |
| GDhz16 | | | | PEDV | | CHN | | 2020 | | MN368686.1 | |  |
| GDhy16 | | | | PEDV | | CHN | | 2020 | | MN368685.1 | |  |
| GDfs16 | | | | PEDV | | CHN | | 2020 | | MN368684.1 | |  |
| FJqz16 | | | | PEDV | | CHN | | 2020 | | MN368683.1 | |  |
| GDzq16 | | | | PEDV | | CHN | | 2020 | | MN368682.1 | |  |
| JXgz15 | | | | PEDV | | CHN | | 2020 | | MN368681.1 | |  |
| HBhg15 | | | | PEDV | | CHN | | 2020 | | MN368680.1 | |  |
| GZgy15 | | | | PEDV | | CHN | | 2020 | | MN368679.1 | |  |
| GXnn15 | | | | PEDV | | CHN | | 2020 | | MN368678.1 | |  |
| GDzq15-3 | | | | PEDV | | CHN | | 2020 | | MN368677.1 | |  |
| GDzq15-2 | | | | PEDV | | CHN | | 2020 | | MN368676.1 | |  |
| GDzq15-1 | | | | PEDV | | CHN | | 2020 | | MN368675.1 | |  |
| GDsg15-3 | | | | PEDV | | CHN | | 2020 | | MN368674.1 | |  |
| GDsg15-2 | | | | PEDV | | CHN | | 2020 | | MN368673.1 | |  |
| GDsg15-1 | | | | PEDV | | CHN | | 2020 | | MN368672.1 | |  |
| GDmm15 | | | | PEDV | | CHN | | 2020 | | MN368671.1 | |  |
| GDjm15 | | | | PEDV | | CHN | | 2020 | | MN368670.1 | |  |
| GDhz15 | | | | PEDV | | CHN | | 2020 | | MN368669.1 | |  |
| GDhy15 | | | | PEDV | | CHN | | 2020 | | MN368668.1 | |  |
| GDgz15-2 | | PEDV | | CHN | | 2020 | | MN368667.1 | |  |  |  |
| GDgz15-1 | | PEDV | | CHN | | 2020 | | MN368666.1 | |  |  |  |
| FJzz15 | | | | | PEDV | | CHN | | 2020 | | MN368665.1 | |
| FJqz15 | | | | | PEDV | | CHN | | 2020 | | MN368664.1 | |
| FJly15 | | | | | PEDV | | CHN | | 2020 | | MN368663.1 | |
| JXyc15 | | | | | PEDV | | CHN | | 2020 | | MN368662.1 | |
| S2-GD2018 | | | | | PEDV | | CHN | | 2018 | | MK533009.1 | |
| V15-HB2018 | | | | | PEDV | | CHN | | 2018 | | MK533010.1 | |
| Q2-AH2017 | | | | | PEDV | | CHN | | 2017 | | MK533008.1 | |
| PB1-SD2018 | | | | | PEDV | | CHN | | 2018 | | MK533007.1 | |
| PA4-GD2018 | | | | | PEDV | | CHN | | 2018 | | MK533006.1 | |
| P13-HB2017 | | | | | PEDV | | CHN | | 2017 | | MK533005.1 | |
| N13-GD2017 | | | | | PEDV | | CHN | | 2017 | | MK533004.1 | |
| N12-GD2017 | | | | | PEDV | | CHN | | 2017 | | MK533003.1 | |
| KGS/KS10P/14 | | | | | PEDV | | Japan | | 2016 | | LC113927.1 | |
| KGS/KS3T/14 | | | | | PEDV | | Japan | | 2016 | | LC113926.1 | |
| 14JM-168 | | | | | PEDV | | Japan | | 2015 | | KM359751.1 | |
| MB-021 | | | | | PEDV | | Canada | | 2014 | | KM196111.1 | |
| ON-007 | | | | | PEDV | | Canada | | 2014 | | KM189366.1 | |
| PEI-023 | | | | | PEDV | | | Canada | 2014 | | | KM189368.1 |
| 2014-022 | | | | | PEDV | | | Canada | 2014 | | | KM196110.1 |
| IC05TK | | | | | PEDV | | | Korea | 2016 | | | KJ857457.1 |
| CJ98 | | | | | PEDV | | | Korea | 2016 | | | KJ857456.1 |
| SM98-5P | | | | | PEDV | | | Korea | 2016 | | | KJ857455.1 |
