# Supplementary material for: Investigation of Transmission and Evolution of PEDV Variants and Co-Infections in Northeast China from 2011 to 2022
Source: Animals (Basel). 2024 Jul 25;14(15):2168. doi: 10.3390/ani14152168 (PMC11311072; doi:10.3390/ani14152168)
Supplement: Supplementary file 1 [file animals-14-02168-s001.zip › Table S2.docx]

**Table S2.** The primers for detection in this study

| **Primer name** | **Target gene** | **Primer sequence (5’**-**3’)** | **Length** | **Reference** | **Use** |
| --- | --- | --- | --- | --- | --- |
| PEDV-S-F | S | ATCGTCAGAGGCATTTTTAA | 4544 bp | This study | Sequencing primers |
| PEDV-S - R |  | TTGGACAGCATCCAAAGACA |  |  |  |
| PEDV- Outer -F | ORF1a | TCTGTAAAACCCGTCACTCTC | 1970bp | This study | Identifying primer |
| PEDV- Outer -R |  | GAGCTCACAAGCAGACTTA |  |  |  |
| PEDV-Inter-F |  | AAGTGCTGTGCTGTCCTCTAG | 1857bp |  | Identifying primer |
| PEDV-Inter-R |  | TCTGTAAAACCCGTCACTCTC |  |  |  |
| PTV-Outer-F | 5'-NTR | AGTTTTGGATTATCTTGTGCCC | 321 bp | Zell et al. (2000) | Identifying primer |
| PTV-Outer-R |  | CCAGCCGCGACCCTGTCAGGCAGCAC |  |  |  |
| PTV-Inter-F |  | TGAAAGACCTGCTCTGGCGCGAG | 158 bp |  | Identifying primer |
| PTV-Inter-R |  | GCTGGTGGGCCCCAGAGAAATCTC |  |  |  |
| TGEV-Outer-F | N | CTTGGTAGTCGTGGTGCTA | 529 bp | Su et al. (2019) | Identifying primer |
| TGEV-Outer-R |  | CTATCTGGTCGCCATCTTC |  |  |  |
| TGEV-Inter-F |  | GCTGTTCTTGCCGCACTTA | 329 bp |  | Identifying primer |
| TGEV-Inter-R |  | ATCTGGTCGCCATCTTCCT |  |  |  |
| PSV-Outer-F | 3C | CCCTGGGACGAAAGAGCCTG | 383 bp | Zell et al. (2000) | Identifying primer |
| PSV-Outer-R |  | CCTTTAAGTAAGTAGTAAAGGG |  |  |  |
| PSV-Inter-F |  | CCAAGATTAGAAGTTGATTTG | 221 bp |  | Identifying primer |
| PSV-Inter-R |  | GGGTAGCCTGCTGATGTAGTC |  |  |  |
| PEV-Outer-F | 5'-NTR | GTACCTTTGTACGCCTGTTTTA | 491 bp | Zell et al. (2000) | Identifying primer |
| PEV-Outer-R |  | ACCCAAAGTAGTCGGTTCCGC |  |  |  |
| PEV-Inter-F |  | CAAGCACTTCTGTTTCCCCGG | 313 bp |  | Identifying primer |
| PEV-Inter-R |  | GTTAGGATTAGCCGCATTCA |  |  |  |
| PCV-Outer-F | URT | ACCAGCGCACTTCGGCAGCGGCAGC | 441 bp | In this study | Identifying primer |
| PCV--Outer-R |  | TCCCGCTCTCCAACAAGGTACTCAC |  |  |  |
| PCV--Inter-F |  | AGGTGGGTGTTCACGCTGAATAATC | 309 bp |  | Identifying primer |
| PCV--Inter-R |  | GTCACTCCGTTGTCCTTGAGATCTA |  |  |  |
| PAstV-Outer-F1 | ORF1b | GARTTYGATTGGRCKCGKTAYGA | 422 bp | Chu et al. (2008) | Identifying primer |
| PAstV-Outer-F2 |  | GARTTYGATTGGRCKAGGTAYGA |  |  |  |
| PAstV-Inter-F1 |  | CGKTAYGATGGKACKATNCC |  |  | Identifying primer |
| PAstV-Inter-F2 |  | AGGTAYGATGGKACKATNCC |  |  |  |
| PoRV-Outer-F | URT | ACCCTTGTACGGGCGCGTCGGGGTT | 383 bp | This study | Identifying primer |
| PoRV -Outer-R |  | AGAAGCTCTCGTCGAAGGCAACTCT |  |  |  |
| PoRV -Inter-F |  | CCGGCGGGCGGGCGCGCCCCGTGGC | 284bp |  | Identifying primer |
| PoRV -Inter-R |  | GAGTGGCGACTCTAGGCAGACGCGC |  |  |  |
| TTSUV-2-Outer-F | URT | AGTTACACATAACCACCAAACC | 264 bp | Wang et al., (2009) | Identifying primer |
| TTSUV-2-Outer-R |  | ATTACCGCCTGCCCGATAGGC |  |  |  |
| TTSUV-2-Inter-F |  | CCAAACCACAGGAAACTGTGC | 225 bp |  | Identifying primer |
| TTSUV-2-Inter-R |  | CTTGACTCCGCTCTCAGGAG |  |  |  |
| PBoV G1-Outer-F | VP1 | AATACCCATACTCACAAAG | 531 bp | Zheng et al. (2016) | Identifying primer |
| PBoV G1-Outer-R |  | GTGATTGATTCATTGCTG |  |  |  |
| PBoV G1-Inter-F |  | ATGTCCCTTATTCTTTCTTG | 268 bp |  | Identifying primer |
| PBoV G1-Inter-R |  | TGATTGGTGTCCGTTTAGT |  |  |  |
| PBoV G2-Outer-F | NP1 | TCCACTGCTTCGAGAACATC | 291 bp | Zheng et al. (2016) | Identifying primer |
| PBoV G2-Outer-R |  | TTCCCTGACATCTTTCCATT |  |  |  |
| PBoV G2-Inter-F |  | CGAGTGACTTCAGCGACAT | 161 bp |  | Identifying primer |
| PBoV G2-Inter-R |  | TGCCAGTAGAAGCCACAAT |  |  |  |
| PBoV G3-Outer-F | NP1/VP1 | GAAATGCTAGAAGCTGTTGA | 384 bp | Zheng et al. (2016) | Identifying primer |
| PBoV G3-Outer-R |  | TACAGGTGACGTTTATTGC |  |  |  |
| PBoV G3-Inter-F |  | GTGTTCTGTTTCCTGGTTAT | 274 bp |  | Identifying primer |
| PBoV G3-Inter-R |  | GTGTCTTTAGGCTCGTTGA |  |  |  |
| PKV-Outer-F | 3D | GGTGGWCTYATHGASTACATGC | 622 bp | [Van](https://www.ncbi.nlm.nih.gov/pubmed/?term=Van%20Dung%20N%5BAuthor%5D&cauthor=true&cauthor_uid=26653281) et al. (2016) | Identifying primer |
| PKV-Outer-R |  | GTGTCNGGRTCCATSACHGGGTG |  |  |  |
| PKV-Inter-F |  | TGGAYTACAAGTGYTTTGATGC | 207 bp |  | Identifying primer |
| PKV-Inter-R |  | ATGATGGTGTTRAKGATRGARGTG |  |  |  |
| PDCoV-Outer-F | N | TGCTACCTCTCCGATTCCCA | 614 bp | Song et al. (2015) | Identifying primer |
|  |  |  |  |  |  |

| PDCoV-Outer-R | ATCCTGTTTGTCTGCTGGCA |  |  |  |
| --- | --- | --- | --- | --- |
| PDCoV-Inter-F | GACACTGAGAAGACGGGTATGG | 238 bp | Identifying primer |  |
| PDCoV-Inter-R | TAGTTGGTTTGGTAGGTGGCTC |  |  |  |
