# Supplementary material for: Investigation of Transmission and Evolution of PEDV Variants and Co-Infections in Northeast China from 2011 to 2022
Source: Animals (Basel). 2024 Jul 25;14(15):2168. doi: 10.3390/ani14152168 (PMC11311072; doi:10.3390/ani14152168)
Supplement: Supplementary file 1 [file animals-14-02168-s001.zip › Table S3.docx]

**Table S3.** Representative sequences of PEDV strain S proteins from 2011 to 2022 and CV777 strain.

**CV777-strain:**

**(GenBank accession no. AF353511, JN599150.1)**

MTPLIYFWLFLPVLLTLSLPQDVTRCQSTINFRRFFSKFNVQAPAVVVLGGYLPSMNS....SSWYCGTGIETDSGVHGIFLSYIDSGQGFEIGISQEPFDPSGYQLYLHKATNGNTSAIARLRICQFPDNKTLGPTVN.DVTTGRNCLFNKAIPA.LQDGKNIVVGITWDNDRVTVFADKIYHFYIKNDWSRVATRCYNKRSCAMQYVYTPTYYMLNVTSAGEDGIYYEPCTANCSGYAANVFATDSNGHIPEGFSFNNWFLLSNDSTLLHGKVVSNQPLLVNCLWAIPKIYGLGQFFSFNQTMDGVCNGAAAQRAPEALRFNINDTFVILAEGSIVLHTALGTNLSFVCSNSSDPHKAIFTIPLGVTEVPYYCFLKVDTYKSTVYKFLAVLPPTVKEIVITKYGDVYVNGFGYLHLGLLDAVTINFTGHGTDDDVSGFWTVASTNFVDALIEVQGTAIQRILYCDDPVSQLKCSQVSFDLDDGFYPISSRNLLSHEQPISFVTLPSFNDHSFVNITVSAAFGGHSGANLIASDTTINGFSSFCVDTRQFTITLFYNVTNSYGYVSKSQDSNCPFTLQSVNDYLSFSKFCVSTSLLAGACTIDLFGYPEFGSGVKFTSLYFQFTKGELITGTPKPLQGVTDVSFMTLDVCTKYTIYGFKGEGIITLTNSSFLAGVYYTSDSGQLLAFKNVTSGAVYSVTPCSFSEQAAYVDDDIVGVISSLSNSTFNNTRELPGFFYHSNDGSNCTEPVLVYSNIGVCKSGSIGYVPLQDGQVKIAPMVTGNISIPTNFSMSIRTEYLQLYNTPVSVDCVTYVCNGNSRCKQLLTQYTAACKTIESALQLSARLESVEVNSMLTISEEALQLATISSFNGDGYNFTNVLGVSVYDPASGRVVQKGSFIEDLLFNKVVTNGLGTVDEDYKRCSNGRSVADLVCAQYYSGVMVLPGVVDAEKLHMYSASLIGGMALGGLTTAAALPFSHAVQARLNYLALQTDVLQRNQQLLAESFNSAIGNITSAFESVKEAISQTSNGLNTVAHALTKVQEVVNSQGSALTQLTIQLQHNFQAISSSIDDIYSRLDILSADVQVDRLITGRLSALNAFVAQTLTKYTEVQASRKLAQQKVNECVKSQSQRYGFCGGDGEHIFSLVQAAPQGLLFLHTVLVPGDFVNVIAIDGLCVNGDIALTLREPGLVLFTHELQTYTATEYFVSSRRMFEPRKPTVSDFVQIESCVVTYVNLTSDQLPDVIPDYIDVNKTLDEILASLPNRIGPSLPLDVFNATYLNLTGEIADLEQRSESLRNTTEELRSLIYNINNTLVDLEWLNRVETYIKWPWWVWLIIFIVLIFVVSLLVFCCISTGCCGCCGCCGACFSGCCRGPRLQPYEAFEKVHVQ

**2011_PEDV-strain:**

**(GenBank accession no.** KF546802.1，KF546800.1，KF546801.1，JQ638915.1-JQ638924.1，JN543367.1，JN381492.1，JN315706.1**)**

MKSLTYFWLFLPVLSTLSLPQDVTRCSANTNFRRFFSKFNVQAPAVVVLGGYLPIGENQGVNSTWYCAGQHPTANGVHGIFLSHIRGGHGFEIGISQEPFDPSGYQLYLHKATNGNTNATARLRICQFPSIKTLGPTANNDVTTGRNCLFNKAIPAHMSEHS..VVGITWDNDRVTVFSDKIYYFYFKNDWSRVATKCYNSGGCAMQYVYEPTYYMLNVTSAGEDGISYQPCTANCIGYAANVFATEPNGHIPEGFSFNNWFLLSNDSTLVHGKVVSNQPLLVNCLLAIPKIYGLGQFFSFNQTIDGVCNGAAVQRAPEALRFNINDTSVILAEGSIVLHTALGTNFSFVCSNSSDPHL....ATFAIPLGAIQVPYYCFLKVDTYNSTVYKFLAVLPPTVREIVITKYGDVYVNGFGYLHLGLLDAVTINFTGHGTDDDVSGFWTIASTNFVDALIEVQGTAIQRILYCDDPVSQLKCSQVSFDLDDGFYPISSTNLLSHEQPTSFVTLPSFNDHSFVNITVSAAFGGHSGANLIASDTTINGFSSFCVDTRQFTISLFYNVTNSYGYVSKSQDTNCPFTLQSVNDYLSFSKFCVSTSLLASACTIDLFGYPEFGSGVKFTSLYFQFTKGELITGTPKPLEGVTDVSFMTLDVCTKYTIYGFKGEGVITLTNSSFLAGVYYTSDSGQLLAFKNVTSGAVYSVTPCSFSEQAAYVDDDIVGVISSLSSSTFNSTRELPGFFYHSNDGSNCTEPVLVYSNIGVCKSGSIGYVPSQSGQVKIAPTVTGNISIPTNFSMSIRTEYLQLYNTPVSVDCATYVCNGNSRCKQLLTQYTAACKTIESALQLSARLESVEVNSMLTISEEALQLATISSFNGDGYNFTNVLGVSVYDPASGRVVQKRSFIEDLLFNKVVTNGLGTVDEDYKRCSNGRSVADLVCAQYYSGVMVLPGVVDAEKLHMYSASLIGGMVLGGFTSAAALPFSYAVQARLNYLALQTDVLQRNQQLLAESFNSAIGNITSAFESVKEAISQTSKGLNTVAHALTKVQEVVNSQGAALTQLTVQLQHNFQAISSSIDDIYSRLDILSADVQVDRLITGRLSALNAFVAQTLTKYTEVQASRKLAQQKVNECVKSQSQRYGFCGGDGEHIFSLVQAAPQGLLFLHTVLVPGDFVDVIAIAGLCVNDEIALTLREPGLVLFTHELQNHTATEYFVSSRRMFEPRKPTVSDFVQIESCVVTYVNLTRDQLPDVIPDYIDVNKTLDEILASLPNRTGPSLPLDVFNATYLNLTGEIADLEQRSESLRNTTEELQSLIYNINNTLVDLEWLNRVETYIKWPWWVWLIIFIVLIFVVSLLVFCCISTGCCGCCGCCCACFSGCCRGPRLQPYEVFEKVHVQ

**2012_PEDV-strain:**

**(GenBank accession no.** KU133263.1-KU133268.1，KU133249.1，KU133252.1，KU133245.1，KU133240.1，KU133239.1，KP870113.1- KP870119.1，KF840557.1，KJ646585.1，KJ646581.1-KJ646583.1，KF546804.1，KF546803.1**)**

MKSLTYFWLFLPVLSTLSLPQDVTRCPANTNFRRFFSKFNVQAPAVVVLGGYLPIGENQGVNSTWYCAGQHPTASGVHGIFLSHIRGGHGFEIGISQEPFDPSGYQLYLHKATNGNTNATARLRICQFPSIKTLGPTANNDVTTGRNCLFNKAIPAHMSEHS..VVGITWDNDRVTVFSDKIYYFYFKNDWSRVATKCYNSGGCAMQYVYEPTYYMLNVTSAGEDGISYQPCTANCIGYAANVFATGPNGHIPEGFSFNNWFLLSNDSTLVHGKVVSNQPLLVNCLLAIPKIHGLGQFFSFNQTIDGVCNGAAVPRAPEALRFNINDTSVILAEGSIVLHTALGTNFSFVCSNSSDPHL....ATFAIPLGAIQVPYYCFLKVDTYNSTVYKFLAVLPPTVREIVITKYGDVYVNGFGYLHLGLLDAVTINFTGHGTDDDVSGFWTIASTNFVDALIEVQGTAIQRILYCDDPVSQLKCSQVAFDLDDGFYPISSTNLLSHEQPTSFVTLPSFNDHSFVNITVSAAFGGHSGANLIASDTTINGFSSFCVDTRQFTISLFYNVTNSYGYVSKSQDSNCPFTLQSVNDYLSFSKFCVSTSLLASACTIDLFGYPEFGSGVKFTSLYFQFTKGELITGTPIPLEGVTDVSFMTLDVCTKYTIYGFKGEGIITLTNSSFLAGVYYTSDSGQLLAFKNVTSGAVYSVTPCSFSEQAAYVDDDIVGVISSLSNSTFNSTRELPGFFYHSNDGSNCTEPVLVYSNIGVCKSGSIGYVPSQSGQVKIAPTVTGNISIPTNFSMSIRTEYLQLYNTPVSVDCATYVCNGNSRCKQLLTQYIAACKTIESALQLSARLESAEVNSMLTISEEALQLATISSFNGDGYNFTNVLGVSVYDPASGRVVQKRSFIEDLLFNKVVTNGLGTVDEDYKRCSNGRSVADLVCAQYYSGVMVLPGVVDAEKLHMYSASLIGGMVLGGFTAAAALPFSYAVQARLNYLALQTDVLQRNQQLLAESFNSAIGNITSAFESVKEAISQTSKGLNTVAHALTKVQEVVNSQGAALTQLTVQLQHNFQAISSSIDDIYSRLDILSADVQVDRLITGRLSALNAFVAQTLTKYTEVQASRKLAQQKVNECVKSQSQRYGFCGGDGEHIFSLVQAAPQGLLFLHTVLVPGDFVNVIAIAGLCVNDEIALTLREPGLVLFTHELQDHTATEYFVSSRRMYEPRKPTVGDFVQIESCVVTYVNLTRDQLPEVIPDYIDVNKTLDEILASLPNRTGPSLSLDVFNATYLNLTGEIADLEQRSESLRNTTEELQSLIYNINNTLVDLEWLNRVETYIKWPWWVWLIIFIVLIFVVSLLVFCCISTGCCGCCGCCGACFSGCCRGPRLQPYEAFEKVHVQ

**2013_PEDV-strain:**

**(GenBank accession no.** KY828999.1, KX982561.1, KX982564.1, KX982557.1, KX982554.1, KX982553.1, KF468755.1，KF601195.1-KF601201.1 ，KR941555.1，KR941556.1 ，KU133262.1，KU133253.1，KU133250.1，KU133241.1-KU133244.1 ，KP870141.1，KP870140.1 **，**KP870120.1 -KP870130.1， KF840562.1，KF840561.1，KJ646586.1 -KJ646590.1，KJ646579.1，KJ646579.1**)**

MKSLTYFWLFLPVLSTLSLPQDVTRCSANTNFRRFFSKFNVQAPAVVVLGGYLPIGENQGVNSTWYCAGQHPTASGVHGIFVSHIRGGHGFEIGISQEPFDPSGYQLYLHKATNGNTNATARLRICQFPNIKTLGPTANNDVTTGRNCLFNKAIPAHMSEHS..VVGITWDNDRVTVFSDKIYYFYFKNDWSRVATKCYNSGGCAMQYVYEPTYYMLNVTSAGEDGISYQPCTANCIGYAANVFATEPNGHIPEGFSFNNWFLLSNDSTLVHGKVVSNQPLLVNCLLAIPKIYGLGQFFSFNQTIDGVCNGAAVQRAPEALRFNINDTSVILAEGSIVLHTALGTNFSFVCSNSSNPHL....ATFAIPLGATQVPYYCFLKVDTYNSTVYKFLAVLPPTVREIVITKYGDVYVNGFGYLHLGLLDAVTINFTGHGTDDDVSGFWTIASTNFVDALIEVQGTAIQRILYCDDPVSQLKCSQVAFDLDDGFYPISSRNLLSHEQPISFVTPPSFNDHSFVNITVSASFGGHSGANLIASDTTINGFSSFCVDTRQFTISLFYNVTNSYGYVSKSQDSNCPFTLQSVNDYLSFSKFCVSTSLLASACTIDLFGYPEFGSGVKFTSVYFQFTKGELITGTPKPLEGVTDVSFMTLDVCTKYTIYGFKGEGIITLTNSSFLAGVYYTSDSGQLLAFKNVTSGAVYSVTPCSFSEQAAYVDDDIVGVISSLSSSTFNSTRELPGFFYHSNDGSNCTEPVLVYSNIGVCKSGSIGYVPSQSGQVKIAPTVTGNISIPTNFSMSIRTEYLQLYNTPVSVDCATYVCNGNSRCKQLLTQYTAACKTIESALQLSARLESVEVNSMLTISEEALQLATISSFNGDGYNFTNVLGVSVYDPASGRVVKKRSFIEDLLFNKVVTNGLGTVDEDYKRCSNGRSVADLVCAQYYSGVMVLPGVVDAEKLHMYSASLIGGMVLGGFTSAAALPFSYAVQARLNYLALQTDVLQRNQQLLAESFNSAIGNITSAFESVKEAISQTSKGLNTVAHALTKVQEVVNSQGAALTQLTVQLQHNFQAISSSIDDIYSRLDILSADVQVDRLITGRLSALNAFVAQTLTKYTEVQASRKLAQQKVNECVKSQSQRYGFCGGDGEHIFSLVQAAPQGLLFLHTVLVPSDFVDVIAIAGLCVNDEIALTLREPGLVLFTHELQNHTATEYFVSSRRMFEPRKPTVSDFVQIESCVVTYVNLTRDQLPDVIPDYIDVNKTLDEILASLPNRTGPSLPLDVFNATYLNLTGEIADLEQRSESLRNTTEELQSLIYNINNTLVDLEWLNRVETYIKWPWWVWLIIFIVLIFVVSLLVFCCISTGCCGCCGCCCACFSGCCRGPRLQPYEVFEKVHVQ

**2014_PEDV-strain:**

**(GenBank accession no.** KY828991.1 -KY828994.1, KX982568.1-KX982572.1, KX982568.1-KX982572.1, KU975423.1，KU975420.1，KU975410.1-KU975413.1，KU975406.1，KU975405.1，KR941552.1- KR941554.1，KU133234.1-KU133261.1 ，KT428879.1，KT428878.1，KT336490.1 ，KP870131.1-KP870139.1，KM196110.1**)**

MKSLNYFWLFLPVLSTLSLPQDVTRCQSTINFRRFFSKFNVQAPAVVVLGGYLPSMNSQGNVSSWYCGTGLETASGVHGIFLSYIDAGQGFEIGISQEPFDPSGYQLYLHKATNSNHNAIARLRICQFPDNKTLGPTVN.DVTTGRNCLFNKAIPAYMQDRKNMVVGITWDNDRVTVFAHKIYHFYLKNDWSRVATRCYNKRSCAMQYVYTPTYYMLNVTSAGEDGIYYEPCTANCSGYAANVFATDSNGHIPEGFSFNNWFLLSNDSTLLHGKVVSNQPLLVNCLLAIPKIYGLGQFFSFNQTMDGVCNGAAAQRAPEALRFNINDTSVILAEGSIVLHTALGTNLSFVCSNSSDPHL....ATFAIPLGATQVPYYCFLKVDTYNSTVYKFLAVLPPTVREIVITKYGDVYVNGFGYLHLGLLDAVTINFTGHGTDDDVSGFWTIASTNFVDALIEVQGTAIQRILYCDDPVSQLKCSQVAFDLDDGFYPISSRNLLSHEQPISFVTLPSFNDHSFVNITVSASFGGHSGANLIASDTTINGFSSFCVDTRQFTISLFYNVTNSYGYVSKSQDSNCPFTLQSVNDYLSFSKFCVSTSLLASACTIDLFGYPEFGSGVKFTSLYFQFTKGELITGTPKPLEGVTDVSFMTLDVCTKYTIYGFKGEGIITLTNSSFLAGVYYTSDSGQLLAFKNVTSGAVYSVTPCSFSEQAAYVDDDIVGVISSLSSSTFNSTRELPGFFYHSNDGSNCTEPVLVYSNIGVCKSGSIGYVPSQSGQVKIAPTVTGNISIPTNFSMSIRTEYLQLYNTPVSVDCATYVCNGNSRCKQLLTQYTAACKTIESALQLSARLESVEVNSMLTISEEALQLATISSFNGDGYNFTNVLGVSVYDPASGRVVKKRSFIEDLLFNKVVTNGLGTVDEDYKRCSNGRSVADLVCAQYYSGVMVLPGVVDAEKLHMYSASLIGGMVLGGFTSAAALPFSYAVQARLNYLALQTDVLQRNQQLLAESFNSAIGNITSAFESVKEAISQTSKGLNTVAHALTKVQEVVNSQGAALTQLTVQLQHNFQAISSSIDDIYSRLDILSADVQVDRLITGRLSALNAFVAQTLTKYTEVQASRKLAQQKVNECVKSQSQRYGFCGGDGEHIFSLVQAAPQGLLFLHTVLVPSDFVDVIAIAGLCVNDEIALTLREPGLVLFTHELQNHTATEYFVSSRRMFEPRKPTVSDFVQIESCVVTYVNLTRDQLPDVIPDYIDVNKTLDEILASLPNRTGPSLPLDVFNATYLNLTGEIADLEQRSESLRNTTEELQSLIYNINNTLVDLEWLNRVETYIKWPWWVWLIIFIVLIFVVSLLVFCCISTGCCGCCGCCCACFSGCCRGPRLQPYEVFEKVHVQ

**2015_PEDV-strain:**

**(GenBank accession no.** KY828996.1, KY828995.1, KX982576.1,KX982573.1, KX982576.1, KX982573.1, KU975422.1，KU975414.1- KU975419.1，KU975409.1，KU975401.1-KU975404.1，MF038016.1，MF038007.1，MF038004.1，MF038003.1，KU133265.1，KU133246.1，KU133235.1，KU133233.1，KU133232.1，MW915428.1-MW915432.1**)**

MKSLTYFWLFLPVLSTLSLPQDVTRCSANTNFRRFFSKFNVQAPAVVVLGGYLPIGENQGVNSTWYCAGQHPTASGVHGIFVSHIRGGHGFEIGISQEPFDPSGYQLYLHKATNGNTNATARLRICQFPSIKTLGPTANNDVTTGLNCLFNKAIPAYMSEHSVFGVTWDNDRVTVFADKIYYFYFKNDWSRVATKCYNSGGCAMQYVYEPTYYMLNVTSAGEGGISYQLCTANCIGYASNVFATQPNGHIPEGFSFNNWFLLSNDSTLVHGNVVSNQPLLVNCLLAIPKIYGLGQFFSFNQTIDGVCNGAAVQRAPEALRFNINDTSVILAEGSIVLHTALGTNFSFVCSNSSNPHLATFAIPLGATQVPYYCFLKVDTYNTPVYKFLAVLPPTVREIVITKYGDVYVNGFGYLHLGLLDAVTINFTSHGTDDDVSGFWTIASTNFVDALIEVQGTAIQRILYCDDPVSQLKCSQVAFDLDDGFYPISSTNLLSHEQPTSFVTLPSFNDHSFVNITVSAAFGGHSGANLIASDTTINGFSSFCVDTRQFTISLFYNVTNSYGYVSNSQDSNCPFTLQSVNDYLSFSKFCVSTSLLASACTIDLFGYPEFGSGVKFTSLYFQFTKGELITGTPKPLEGVTDVSFMTLDVCTKYTIYGFKGEGIITLTNSSFLAGVHYTSDSGQLLAFKNVTSGAVYSVTPCSFSEQAAYVDDDIVGVISSLSNSTFNSTRELPGFFYHSNDGSNCTEPVLVYSNIGVCKSGSIGYVSSQSGQVKIAPTVTGNISIPTNFSMSIRTEYLQLYNTPVSVDCATYVCNGNSRCKQLLTQYTSACKTIESALQLSARLESAEVNSMLTISEEALQLATISSFNGDGYNFTNVLGVSVYDPASGRVVQKRSFIEDLLFNKVVTNGLGTVDEDYKRCSNGRSVADLVCAQYYSGVMVLPGVVDAEKLHMYSASLIGGMVLGGFTAAAALPFSYAVQARLNYLALQTDVLQRNQQLLAESFNSAIGNITSAFESVKEAISQTSKGLNTVAHALTKVQEVVNSQGAALTQLTVQLQHNFQAISSSIDDIYSRLDILSADAQVDRLITGRLSALNAFVAQTLTKYTEVQASRKLAQQKVDECVKSQSQRYGFCGGDGEHIFSLVQAAPQGLLFLHTVLVPGDFVNVIAIAGLCVNDEIALTLREPGLVLFTHELQDTATEYFVSSRRMYEPRKPTVGDFVQIESCVVTYVNLTRDQLPEVIPDYIDVNKTFDEILASLPNRTGPSLSLDVFNATYLNLTGEIADLEHRSESLRNTTEELRSLIYNINNTLVDLEWLNRVETYIKWPWWVWLVIFIVLIFVVSLLVFCCISTGCCGCCGCCGACFSGCCRGPRLQPYEAFEKVHVQ

**2016_PEDV-strain:**

**(GenBank accession no.** KY828998.1, KY828997.1, LC496368.1, MG020538.1 -MG020555.1**)**

MRSLTYFWLFLPVLSTFSLPQDVTRCSANTNFRRFFSKFNVQAPAVVVLGGYLPTGENQGVNSTWYCAGQHSTASGVHGIFLSHIRGGHGFEIGISQEPFDSSGYQLYLHKATNGNTNATARLRICQFPSIKTLGPTANNDVTTGRNCLFNKAIPAHMSEHS..VVGITWDNDRVTVFSDKIYHFYFKNDWSRVATKCYNSGGCAMQYVYEPTYYMLNVTSAGEDGISYQPCTANCIGYAANVFATEPNGHIPEGFSFNNWFLLSNDSTVLHGKVVSNQPLLVNCLLAIPKIYGLGQFFSFNQTMDGVCNGAAAQRAPEALRFNINDTSVILAEGSIVLHTALGTNLSFVCSNSSDPHS....AIFAIPLGATQVPYYCFLKVDTYNSTVYKFLAVLPPTVREIVITKYGDVYVNGFGYLHLGLLDAVTINFTGHRTDGDVSGFWTIASTNFVDALIEVQGTAIQRILYCDDPVSQLKCSQVAFDLDDGFYPISSINLLSHEQSTSFVTLPSFNDHSFVNITVSAAFGGHSGANLIASDTTINGFSSFCVDTRQFTITLFYNVTNSYGYVSKSQDSNCPFTLQSVNDYLSFSKFCVSTSLLASACTIDLFGYPEFGSGVKFTSLYFQFTKGELITGTPKPLEGVTDVSFMTLDVCTKYTIYGFKGEGIITLTNSSFLAGVYYTSDSGQLLAFKNVTSGAIYSVTPCSFSEQAAYVDDDIVGVISSLSSSTFNNTRELPGFFYHSNDGSNCTEPVLVYSNIGVCKSGSIGYVSSQSGQVKIAPTVTGNISIPTNFSMSIRTEYLQLYNTPVSVDCATYVCNGNSRCKQLLTQYTAACKTIESALQLSARLESAEVNSMLTISEEALQLATISSFNGDGYNFTNVLGVSVYDPASGRVVQKRSFIEDLLFNKVVTNGLGTVDEDYKRCSNGRSVADLVCAQYYSGVMVLPGVVDAEKLHMYSASLIGGMVLGGFTAAAALPFSYAVQARLNYLALQTDVLQRNQQLLAESFNSAIGNITSAFESVKDAISQTSKGLNTVAHALTKVQEVVNSQGAALTQLTVQLQHNFKAISSSIDDIYSRLDILSADVQVDRLITGRLSALNAFVAQTLTKYTEVQASRKLAQQKVNECVKSQSQRYGFCGGDGEHIFSLVQAAPQGLLFLHTVLVPGDFVNVIAIAGLCVNDEIALTLREPGLVLFTHELQD.TATEYFVSSRRMYEPRKPTVGDFVQIESCVVTYVNLTRDQLPEVIPDYIDVNKTLDEILASLPNRTGPSLSLDVFNATYLNLTGEIADLEQRSESLRNTTEELQSLIYNINNTLVDLEWLNRVETYIKWPWWVWLIIFIVLIFVVSLLVFCCISTGCCGCCGCCGACFSGCCRGPRLQPYEAFEKVHVQ

**2017_PEDV-strain:**

**(GenBank accession no.** MH593138.1，MH053412.1-MH053419.1，MW915433.1-MW915437.1，MW915418.1-MW915427.1，MW826593.1-MW826599.1，MK533008.1，MK533005.1，MK533003.1**)**

MKSLTYFWLFFPLLSTLSLPQDVTRCQLTTNFRRFFSKFNVQAPAVVVLGGYLPSMNSQGNVSSWYCGTGLETASGVHGIFLSYIDSGQGFEIGISQEPFDPSGYQLYLHKATNGNTGAVARLRICQFPDNKTLGPSS..GVTSGRNCLFNKAIPAHMQDGKNIVIGITWDNDRVTVFADKIYHFYLKNDWSRVATRCYNKRSCAMQYVYTPTYYMLNVTSAGEDGIYYEPCTANCIGYAANVFATEPNGHIPEGFSFNNWFLLSNDSTLVHGKVVSNQPLLVNCLLAIPKIYGLGQFFSFNQTIDGVCNGAAVQRAPEALRFNTNDTSVILAEGSIVLHTAFGTNLSFVCSNSSNPHL....ATFAIPLGATQVTYYCFLKVDTYNSTVYKFLAVLPPTVREIVITKYGDVYVNGFGYLHIGLLDAVTINFTGHGTDDDVSGFWTIASTNFVDALIEVQGTAIQRILYCDDPVSQLKCSQVAFDLDDGFYPISSRNLLSHEQPISFVTLPSFNDHSFVNITVSASFGGHSGANLIASDTTINGFSSFCVDTRQFTISLFYNVTNSYGYVSKSQDSNCPFTLQSVNDYLSFSKFCVSTSLLASACTIDLFGYPEFGSGVKFTSLYFQFTKGELITGTPKPLEGVTDVSFMTLDVCTKYTIYGFKGEGIITLTNSSFLAGVYYTSDSGQLLAFKNVTSGAVYSVTPCSFSEQAAYVDDDIVGVISSLSSSTFNSTRELPGFFYHSNDGSNCTEPVLVYSNIGVCKSGSIGYVPSQSGQVKIAPTVTGNISIPTNFSMSIRTEYLQLYNTPVSVDCATYVCNGNSRCKQLLTQYTSACKTIESALQLSARLESVEVNSMLTISEEALQLATISSFNGDGYNFTNVLGVSVYDPASGRVVQKRSFIEDLLFNKVVTNGLGTVDEDYKRCSNGRSVADLVCAQYYSGVMVLPGVVDAEKLHMYSASLIGGMVLGGFTSAAALPFSYAVQARLNYLALQTDVLQRNQQLLAESFNSAIGNITSAFESVKEAISQTSKGLNTVAHALTKVQEVVNSQGAALTQLTVQLQHNFQAISSSIDDIYSRLDILSADVQVDRLITGRLSALNAFVAQTLTKYTEVQASRKLALQKVNECVKSQSQRYGFCGGDGEHIFSLVQAAPQGLLFLHTVLVPGDFVDVIAVAGLCVNDEIALTLREPGLVLFTHELQNHTATEYFVSSRRMFEPRKPTVSDFVQIESCVVTYVNLTRDQLPDVIPDYIDVNKTLDEILASLPNRTGPSLPLDVFNATYLNLTGEIADLEQRSESLRNTTEELQSLIYNINNTLVDLEWLNRVETYIKWPWWVWLIIFIALIFVVSLLVFCCISTGCCGCCGCCCACFSGCCRGPRLQPYEVFEKVHVQ

**2018_PEDV-strain:**

**(GenBank accession no.** >MT294128.1- MT294135.1， MN617858.1-MN617867.1，MK592415.1- MK598821.1 ，MH593153.1，MH678638.1，MH593139.1-MH593152.1，MH053420.1，MH053413.1 ，MT031818.1-MT031820.1，MK533006.1-MK533010.1 **)**

MKSLTYFWLFLPVLSTLSLPQDVTRCSANTNFRRFFSKFNVQAPAVVVLGGYLPIGENQGVNSTWYCAGQHPTASGVHGIFLSHIRGGHGFEIGISQEPFDPSGYQLYLHKATNGNTNATARLRICQFPSIKTLGPTADNDVTTGRNCLFNKAIPAHMSEHS..VVGITWDNDRVTVFSDKIYHFYFKNDWSRVATKCYNSGGCAMQYVYEPTYYMLNVTSAGEDGISYQLCTANCIGYAANVFATEPNGHIPEGFSFNNWFLLSNDSTLVHGKVVSNQPLLVNCLLAMPKIYGLGQFFSFNQTIDGVCNGAAVQRAPEALRFNINDTSVILAEGSIVLHTALGTNLSFVCSNSSDPHL....ATFAIPLGATQVPYYCFLKVDTYNSTVYKFLAVLPPTVREIVITKYGDVYVNGFGYLHLGLLDAVTINFTGHGTDDDVSGFWTIASTNFVDALIEVQGTAIQRILYCDDPVSQLKCSQVAFDLDDGFYPISSRNLLSHEQPISFVTLPSFNDHSFVNITVSASFGGHSGANLIASDTTINGLSSFCVDTRQFTISLFYNVTNSYGYVSKSQDSNCPFTLQSVNDYLSFSKFCVSTSLLASACTIDLFGYPEFGSGVKFTSLYFQFTKGELITGTPKPLEGVTDVSFMTLDVCTKYTIYGFKGEGIITLTNSSILAGVYYTSDSGQLLAFKNVTSGAVYSVTPCSFSEQAAYVDDDIVGVISSLSSSTFNSTRELPGFFYHSNDGSNCTEPVLVYSNIGVCKSGSIGYVPSQSGQVKIAPTVTGNISIPTNFSMSIRTEYLQLYNTPVSVDCATYVCNGNSRCKQLLTQYTAACKTIESALQLSARLESVEVNSMLTISEEALQLATISSFNGDGYNFTNVLGVSVYDPASGRVVQKRSFIEDLLFNKVVTNGLGTVDEDYKRCSNGRSVADLVCAQYYSGVMVLPGVVDAEKLHMYSASLIGGMVLGGFTSAAALPFSYAVQARLNYLALQTDVLQRNQQLLAESFNSAIGNITSAFESVKEAISQTSKGLNTVAHALTKVQEVVNSQGAALTQLTVQLQHNFQAISSSIDDIYSRLDILSADVQVDRLITGRLSALNAFVAQTLTKYTEVQASRKLAQQKVNECVKSQSQRYGFCGGDGEHIFSLVQAAPQGLLFLHTVLVPGDFVNVIAIAGLCVNDEIALTLREPGLVLFTHELQD.TATEYFVSSRRMYEPRKPTVGDFVQIESCVVTYVNLTRDQLPEVIPDYIDVNKTLDEILASLPNRTGPSLSLDVFNATYLNLTGEIADLEQRSESLRNTTEELQSLIYNINNTLVDLEWLNRVETYIKWPWWVWLIIFIVLIFVVSLLVFCCISTGCCGCCGCCGACFSGCCRGPRLQPYEAFEKVHVQ

**2019_PEDV-strain:**

**(GenBank accession no.** MT090137.1**-**MT090140.1, MN161584.1，MK820037.1-MK820042.1，MK685665.1 ，MW145531.1，MW145530.1 **)**

MKSLTYFWLFLPVLSTLSLPQDVTRCSANTNFRRFFSKFNVQAPAVVVLGGYLPIGENQGVNSTWYCAGQHPTASGVHGIFVSHIRGGHGFEIGISQEPFDPSGYQLYLHKATNGNTNATARLRICQFPNIKTLGPTANNDVTTGRNCLFNKAIPAYMSEHS..VVGITWDNDRVTVFSDKIYHFYFKNDWSRVATRCYNSGGCAMQYVYEPTYYMLNVTSGGEDGISYQPCTANCIGYSANVFATEPNGHIPEGFSFNNWFLLSNDSTLAHGKVVSNQPLLVNCLLTTPKIYGLGQFFSFNQTIDGVCNGAAVQRAPEALRFNINDTSVILAEGSIVLHTALGTNLSFVCSNSSNPHL....ATFAIPLGATQVPYYCFLKVDTYNSTVYKFFAVLPPTVREIVITKYGDVYVNGFGYLHLGLLDAVTINFTGHGTDDDVSGFWTIASTTFVDALIEVQGTAIQRILYCDDPVSQLKCSQVSFDLDDGFYPISSRNLLSHEQPISFVTLPSFNDHSFVNITVSATFGGHSGANLIASDTTINGFSSFCVDTRQFTISLFYNVTNSYGYVSKSQDSNCPFTLQSVNDYLSFSKFCVSTSLLASACTIDLFGYPEFGSGVKFTSLYFQFTKGELITGTPKPLEGVTDVSFMTLDVCTKYTIYGFKGEGIITLTNSSFLAGVYYTSDSGQLLAFKNVTSGAVYSVTPCSFSEQAAYVDDDIVGVISSLSSSTFNSTRELPGFFYHSNDGSNCTEPVLVYSNIGVCKSGSIGYVPSQSGQVKIAPTVTGNISIPTNFSMSIRTEYLQLYNTPVSVDCATYVCNGNSRCKQLLTQYTAACKTIESALQLSARLESVEVNSMLTISEEALQLATISSFNGDGYNFTNVLGASVYDPASGRVVQKRSFIEDLLFNKVVTNGLGTVDEDYKRCSNGRSVADLVCAQYYSGVMVLPGVVDAEKLHMYSASLIGGMVLGGFTAAAALPFSYAVQARLNYLALQTDVLQRNQQLLAESFNSAIGNITSAFESVKEAISQTSKGLNTVAHALTKVQEVVNSQGAALTQLTVQLQHNFQAISSSIDDIYSRLDILSADVQVDRLITGRLSALNAFVSQTLTKYTEVQASRKLAQQKVNECVKSQSQRYGFCGGDGEHIFSLVQAAPQGLLFLHTVLVPGDFVDVIAIAGLCVNDEIALTLREPGLVLFTHELQNHTATEYFVSSRRMFEPRKPTVSDFVQIESCVVTYVNLTRDQLPDVIPDYIDVNKTLDEILSSLPNRTGPSLPLDVFNATYLNLTGEIADLEQRSESLRNTTEELQSLIYNINNTLVDLEWLNRVETYIKWPWWVWLIIFIVLIFVVSLLVFCCISTGCCGCCGCCCACFSGCCRGPRLQPYEVFEKVHVQ

**2020_PEDV-strain:**

**(GenBank accession no.** MZ161077.1-MZ161089.1 **，** MZ160999.1-MZ161066.1 **)**

MKSLTYFWLFLPVLSTLSLPQDVTRCSANTNFRRFFSKFNVQAPAVVVLGGYLPIGENQGVNSTWYCAGQHPTASGVHGIFLSHIRGGHGFEIGISQEPFDPSGYQLYLHKATNGNTNATARLRICQFPSIKTLGPTADNDVTTGRNCLFNKAIPAHMSEHS..VVGITWDNDRVTVFSDKIYHFYFKNDWSRVATKCYNSGGCAMQYVYEPTYYMLNVTSAGEDGISYQLCTANCIGYAANVFATEPNGHIPEGFSFNNWFLLSNDSTLVHGKVVSNQPLLVNCLLAMPKIYGLVQFFSFNQTIDGVCNGAAVQRAPEALRFNINDTSVILAEGSIVLHTALGTNLSFVCSNSSDPHL....ATFAIPLGATQVPYYCFLKVDTYNSTVYKFLAVLPPTVREIVITKYGDVYVNGFGYLHLGLLDAVTINFTGHGTDDDVSGFWTIASTNFVDALIEVQGTAIQRILYCDDPVSQLKCSQVAFDLDDGFYPISSRNLLSHEQPISFVTLPSFNDHSFVNITVSASFGGHSGANLIASDTTINGLSSFCVDTRQFTISLFYNVTNSYGYVSKSQDSNCPFTLQSVNDYLSFSKFCVSTSLLASACTIDLFGYPEFGSGVKFTSLYFQFTKGELITGTPKPLEGVTDVSFMTLDVCTKYTIYGFKGEGIITLTNSSILAGVYYTSDSGQLLAFKNVTSGAVYSVTPCSFSEQAAYVDDDIVGVISSLSSSTFNSTRELPGFFYHSNDGSNCTEPVLVYSNIGVCKSGSIGYVPSQSGQVKIAPTVTGNISIPTNFSMSIRTEYLQLYNTPVSVDCATYVCNGNSRCKQLLTQYTAACKTIESALQLSARLESVEVNSMLTISEEALQLATISSFNGDGYNFTNVLGVSVYDPASGRVVQKRSFIEDLLFNKVVTNGLGTVDEDYKRCSNGRSVADLVCAQYYSGVMVLPGVVDAEKLHMYSASLIGGMVLGGFTSAAALPFSYAVQARLNYLALQTDVLQRNQQLLAESFNSAIGNITSAFESVKEAISQTSKGLNTVAHALTKVQEVVNSQGAALTQLTVQLQHNFQAISSSIDDIYSRLDILSADVQVDRLITGRLSALNAFVAQTLTKYTEVQASRKLAQQKVNECVKSQSQRYGFCGGDGEHIFSLVQAAPQGLLFLHTVLVPGDFVDVIAIAGLCVNDEIALTLREPGLVLFTHELQNHTATEYFVSSRRMFEPRKPTVSDFVQIESCVVTYVNLTRDQLPDVIPDYIDVNKTLDEILASLPNRTGPSLPLDVFNATYLNLTGEIADLEQRSESLRNTTEELQSLIYNINNTLVDLEWLNRVETYIKWPWWVWLIIFIVLIFVVSLLVFCCISTGCCGCCGCCCACFSGCCRGPRLQPYEVFEKVHVQ

**2021_PEDV-strain:**

**(GenBank accession no.** UBF40533.1，MZ161067.1-MZ161076.1 **)**

MKSLTYFWLFLPVLSTFSLPQDVTRCQLTTNFRRFFSKFNVQAPAVVVLGGYLPSMNSQGNVSSWYCGTGLETASGVHGIFLSYIDSGQGFEIGISQEPFDPSGYQLYLHKATNGNIGAVARLRICQFPDNKTLGPSS..GVTSGRNCLFNKAIPAHMQDGKNVVIGITWDNDRVIVFADKIYHFYLKNDWSRVATRCYNKRSCAMQYVYTPTYYMLNVTSAGEDGIYYEPCTANCTGYAANVFATEPNGYIPEGFSFNNWFLLSNDSTLVHGKVVSNQPLLVNCLLAIPKIYGLGQFFSFNQTIDGVCNGASVQRAPEALRFNINATSVILAEGSIVLHTAFGTNLSFVCSNSSNPHL....ATFAIPLGATQVPYYCFLKVDTYNSTVYKFLAVLPPTVREIVITKYGDVYVNGFGYLHIGLLDAVTINFTGHGTDDDVSGFWTIASTNFVDALIEVQGTAIQRILYCDDPVSQLKCSQVAFDLDDGFYPISSRNFLSHEQPISFVTLPSFNDHSFVNITVSASFGGHSGANLIASDTTINGFSSFCVDTRQFTISLFYNVTNSYGYVSKSQDSNCPFTLQSVNDYLSFSKFCVSTSLLASACTIDLFGYPEFGSGVKFTSLYFQFTKGELITGTPKPLEGVTDVSFMTLDVCTKYTIYGFKGEGIITLTNSSFLAGVYYTSDSGQLLAFKNVTSGAVYSVTPCSFSEQAAYVDDDIVGVISSLSSSTFNSTRELPGFFYHSNDGSNCTEPVLVYSNIGVCKSGSIGYVPSQSGQVKIAPTVTGNISIPTNFSMSIRTEYLQLYNTPVSVDCATYVCNGNSRCKQLLTQYTAACKTIESALQLSARLESVEVNSMLTISEEALQLATISSFNGDGYNFTNVLGVSVYDPASGRVVQKRSFIEDLLFNKVVTNGLGTVDEDYKRCSNGRSVADLVCAQYYSGVMVLPGVVDAEKLHMYSASLIGGMVLGGFTSAAALPFSYAVQARLNYLALQTDVLQRNQQMLAESFNSAIGNITSAFESVKEAISQTSKGLNTVAHALTKVQEVVNSQGAALTQLTVQLQHNFQAISSSIDDIYSRLDILSADVQVDRLITGRLSALNAFVAQTLTKYTEVQASRKLAQQKVNECVKSQSQRYGFCGGDGEHIFSLVQAAPQGLLFLHTVLVPGDFVDVIAIAGLCVNDEIALTLREPGLVLFTHELQNHTATEYFVSSRRMFEPRKPTVSDFVQIESCVVTYVNLTRDQLPDVIPDYIDVNKTLDEILASLPNRTGPSLPLDVFNATYLNLTGEIADLEQRSESLRNTTEELQSLIYNINNTLVDLEWLNRVETYIKWPWWVWLIIFIVLIFVVSLLVFCCISTGCCGCCGCCCACFSGCCRGPRLQPYEVFEKVHVQ

**2022_PEDV-strain:**

**(GenBank accession no.**   ON988085.1-ON988096.1，OL944723.1， MW560718.1- MW560718.1**)**

MKSLTYFWLFLPVLSTLSLPQDVTRCSANTNFRRFFSKFNVQAPAVVVLGGYLPIGENQGVNSTWYCAGKHPTASGVHGIFVSHIRGGHGFEIGISQEPFDPSGYQLYLHKATNGNTNATARLRICQFPSIKTLGPTANNDVTTGRNCLFNKAIPAHMSEHS..VVGITWDNDRVTVFSDKIYYFYFKNDWSRVATKCYNSGGCAMQYVYEPTYYMLNVTSAGEDGISYQPCTANCIGYAANVFATEPNGHIPEGFSFNNWFLLSNDSTLVHGKVVSNQPLLVNCLLAIPKIYGLGQFFSFNQTIDGVCNGAAVQRAPEALRFNINDTSVILAEGSIVLHTALGTNFSFVCSNSSDPHLNMRSATFAIPLGAIQVPYYCFFKVDTYNSTVYKFLAVLPPTVREIVITKYGDVYVNGFGYLHLGLLDAVTINFTGHGTDDDVSGFWTIASTNFVDALIEVQGTAIQRILYCDDPVSQLKCSQVAFDLDDGFYPISSRNLLSHEQPISFVTLPSFNDHSFVNITVSASFGGHSGANLIASDTTINGFSSFCVDTRQFTISLFYNVTNSYGYVSKSQDSNCPFTLQSVNDYLSFSKFCVSTSLLASACTIDLFGYPEFGSGVKFTSLYFQFTKGELITGTPKPLEGVTDVSFMTLDVCTKYTIYGFKGEGIITLINSSFLAGVYYTSDSGQLLAFKNVTSGAVYSVTPCSFSEQAAYVDDDIVGVISSLSNSTFNSTRELPGFFYHSNDGSNCTEPVLVYSNIGVCKSGSIGYVPSQSGQVKIAPTVTGNISIPTNFSMSIRTEYLQLYNTPVSVDCATYVCNGNSRCKQLLTQYTAACKTIESALQLSARLESVEVNSMLTISEEALQLATISSFNGDGYNFTNVLGVSVYDPARGRVVQKRSFIEDLLFNKVVTNGLGTVDEDYKRCSNGRSVADLVCAQYYSGVMVLPGVVDAEKLHMYSASLIGGMVLGGITAAAALPFSYAVQARLNYLALQTDVLQRNQQLLAESFNSAIGNITSAFESVKEAISQTSKGLNTVAHALTKVQEVVNSQGAALTQLTVQLQHNFQAISSSIDDIYSRLDILSADVQVDRLITGRLSALNAFVAQTLTKYTEVQASRKLAQQKVNECVKSQSQRYGFCGGDGEHIFSLVQAAPQGLLFLHTVLVPGDFVDVIAIAGLCVNDEIALTLREPGLVLFTHELQNHTATEYFVSSRRMFEPRKPTVGDFVQIESCVVTYVNLTRDQLPDVIPDYIDVNKTLDEILASLPNRTGPSLSLDVFNATYLNLTGEIADLEQRSESLRNTTEELQSLLYNINNTLVDLEWLNRVETYIKWPWWVWLIIFIVLIFVVSLLVFCCISTGCCGCCGCCGACFSGCCRGPRLQPYEAFETVHVQ
