# Supplementary material for: Investigation of Transmission and Evolution of PEDV Variants and Co-Infections in Northeast China from 2011 to 2022
Source: Animals (Basel). 2024 Jul 25;14(15):2168. doi: 10.3390/ani14152168 (PMC11311072; doi:10.3390/ani14152168)
Supplement: Supplementary file 1 [file animals-14-02168-s001.zip › Table S6.docx]

Table S6: Results of correlation chi-square analysis.

| Virus | PKV | PAstV | PBoV | PCV | PDCoV | PEV | PoRV | PSV | PTV | TGEV |
| --- | --- | --- | --- | --- | --- | --- | --- | --- | --- | --- |
| P value | 0.626 | 0.588 | 1.317 | 0.321 | 1.826 | 1.108 | 0.211 | 0.842 | 0.266 | 0.675 |
